# Food sharing networks in a multi-ethnic, predominantly indigenous peri-urban community in the Brazilian Amazon

**DOI:** 10.1371/journal.pone.0360629

**Published:** 2026-11-11

**Authors:** Harold Martin Wright III, Barbara A. Piperata, Sean S. Downey, Shane A. Scaggs, Therezinha de Jesus Pinto Fraxe

**Affiliations:** 1 Department of Anthropology, The Ohio State University, Columbus, Ohio, United States of America; 2 School of Environment and Natural Resources, The Ohio State University, Columbus, Ohio, United States of America; 3 Faculdade de Ciências Agrárias, Universidade Federal do Amazonas, Manaus, Brazil; University of Mississippi, BRAZIL

## Abstract

Social networks have long supported access to food among rural Indigenous and non‑Indigenous agrarian populations. As increasing numbers of rural Indigenous and agrarian households migrate to urban areas, questions arise about how these social networks shape food access in peri‑urban and urban settings where cash‑based markets predominate. Some researchers argue that, as Indigenous and agrarian households migrate to peri‑urban and urban settings, economic capital (“buying power”) becomes the primary means of accessing food, reducing reliance on social capital and food‑sharing networks. Others find that migrants develop new affinity‑based support networks, often with individuals of similar regional or cultural backgrounds, which continue to play an important role in securing food in urban contexts. To better understand peri-urban food sharing, we examine the properties of the food-sharing networks of 106 households in Parque das Tribos, a primarily Indigenous, multiethnic community located in Manaus, Brazil. We utilize regressions to test the predictive relationship between residents’ perceptions of household food security using a locally adapted version of the Brazilian Food Insecurity Survey (EBIA) instrument and social network properties, as well as the role of household socioeconomic factors in predicting their levels of engagement in food-sharing relationships. We find a positive relationship between perception of household food security and participation in larger, higher quality, relatively dense networks based on reciprocal relationships, even after accounting for economic factors such as income and conditional cash transfer programs. Overall, the results indicate that social capital is more strongly associated with household food security than economic capital.

## Introduction

According to the United Nation’s Food and Agricultural Organization FAO [1], “Food security exists when all people, at all times, have physical, social, and economic access to sufficient, safe and nutritious food which meets their dietary needs and food preferences for an active and healthy life.” Food insecurity is, then, the inability to access adequate, nutritious, and culturally appropriate foods in socially acceptable ways [2–5]. An estimated 2.33 billion people, or 29% of the world’s population, experienced moderate or severe food insecurity in 2023 [6]. A large literature demonstrates the negative implications of food insecurity for people’s physical, mental, and social well-being [7–11].

The FAO has identified four “pillars” that support food security: *Availability, Access, Stability (or Reliability),* and *Utilization* [12]. Of these, most household-level research focuses on financial access to food [13]. Thus, researchers regularly associate food security with household economic capital, or “buying power” [14–17]. However, food-sharing relationships have long served as a means of accessing food and continue to be important among Indigenous and rural populations in rural areas where cash economies are limited, mixed, or non-existent, particularly in the Global South and Arctic [5,18–21]. Studies among riverine communities in the Brazilian Amazon [22–24], Maasai communities in Tanzania [18,25], and Indigenous households in Canada [26–29] have examined the role that personal relationships play in resource acquisition, processing, and distribution, as well as how these subsistence activities are shaped by social status and prestige. Among rural Indigenous communities in the Amazon, interethnic and intercommunity food exchanges not only support subsistence, but also enhance the prestige of individuals, families, and sibs, as evidenced among the predominantly exogamous groups of the Northwest Amazon [30–35]. However, it is unclear how these food sharing relationships change after migrating to peri-urban and urban areas. According to the 2022 census, 54% of Brazil’s Indigenous population resides in urban and peri-urban areas, and these numbers are expected to continue growing [36]. Thus, understanding how Indigenous people in urban and peri-urban settings perceive their food security, and how culturally embedded practices such as food sharing and material resource transfers shape these perceptions, is critically important yet remains largely understudied.

The aim of this study is to analyze the role of social networks in alleviating food insecurity by examining associations between social capital, as measured by the social network measures of size, density, and quality, and perception of food security at the household level in a multi-ethnic, predominantly Indigenous peri-urban community.

### Sharing-based social capital

Social capital is one of three types of capital, including cultural capital and economic capital [37,38]. Social capital is characterized by the social obligations established through reciprocal exchanges, both direct (between two parties) and indirect (i.e., “paying it forward”) [39–41]. Fulfilling these obligations results in a positive reputation that increases over time [37,42,43]. This is certainly true among many non-western populations, including many rural and Indigenous peoples, where social networks, including food sharing, remain common [29,44–46]. Food sharing at the interhousehold level is associated with the emergence of complex networks of distribution aimed at protecting vulnerable community members and diminishing risk, particularly in isolated communities. These mechanisms are often hierarchical as expectations and modes of support expand, with friends of friends becoming integrated into one’s personal network, resulting in increased trust among individuals across social and relational scale [27,42,47–49].

### Traditional food-sharing in urban and peri-urban spaces

In the Amazon, peri-urban communities are frequently multi-ethnic and include Indigenous people, as well as non-Indigenous rural migrants whose cultural backgrounds are more similar to those of Indigenous peoples than to urban populations [50–53]. As with Indigenous communities throughout the Americas, these peri‑urban communities often form through homophily, with individuals of similar ethnic, regional, religious, or cultural backgrounds “sticking together” in unfamiliar settings [53–55]. Thus, it is plausible that social networks remain a critical mechanism for enhancing food security in peri-urban and urban communities dominated by migrants from rural areas.

Some studies suggest that access to financial resources can negatively impact social networks, including food-sharing ones, even in rural communities relatively distant from markets [23,27,28,56,57]. They argue that access to monetary resources is frequently associated with the breakdown of social networks, including food sharing networks, as people begin using economic capital over social capital to access food [23,58]. However, others find that monetary resources are associated with greater engagement in social networks as they add a monetary dimension to food access [59–61]. Within peri‑urban, predominantly Indigenous communities, the respective roles of money‑based urban markets and traditional food‑sharing practices raise important questions about how households perceive their food security.

### Network measures and food security

In this study, we analyze *egocentric networks*, which are comprised of the relationships between an “ego” who we interviewed, and his or her “alters” (friends, neighbors, kin, etc.), to examine resource exchanges related to food access. Egocentric networks are well suited for large, complex communities where interviewing all actors is impractical [62–64], as is the case here.

Research on social networks and food access highlights three key measures [22,27,39,65,66]. *Network size* is the total number of connections that an actor has, including incoming and outgoing resource flows [64]. Network quality refers to the strength, reliability, and multiplexity of social ties, not a normative or value‑based assessment of individuals or households. Within food security, actors with multiple independent inward ties, especially *bridge ties*, or connections to alters who themselves are not interconnected, often report access to more varied food sources [67].

*Network density* is the total number of connections between all actors within a network. According to Burt’s concept of structural holes [65], highly dense networks tend to provide strong and steady support, though they may limit diversity due to resource redundancy. Even in less dense networks, support can remain strong when the same alters exchange multiple types of resources, a pattern known as *multiplexity of exchange* [27,65,67,68].

Dense networks can also be large when tightly connected subgroups are linked through singular bridge ties [22,65] (Fig 1). Actors who serve as the sole conduit through which subgroups obtain particular resources often occupy positions of higher prestige, especially when they are connected to other influential individuals [69–71]. This combination of strategic positioning and resource flow is captured by *network quality* [27]*.*

**Fig 1:**
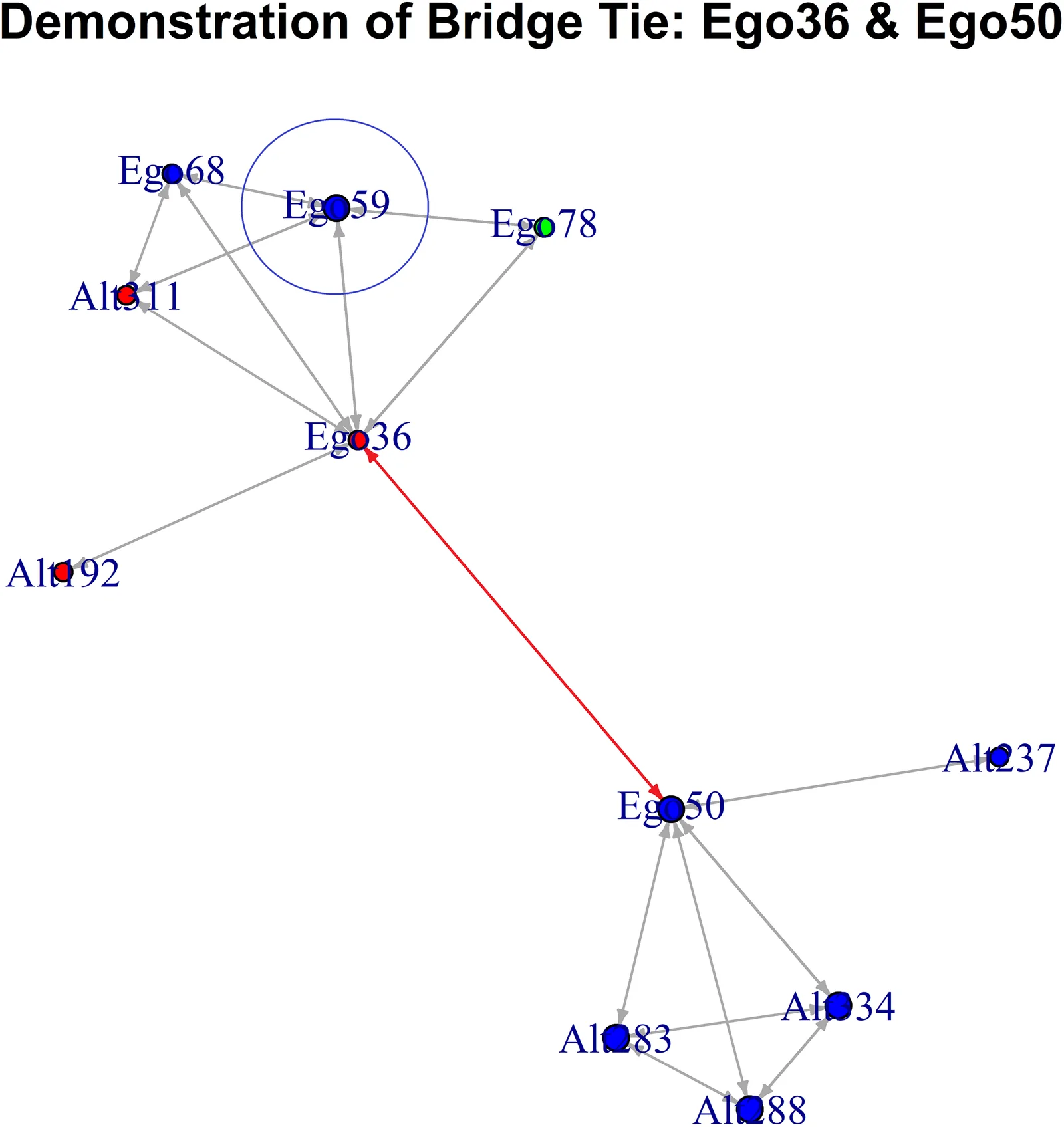
Example of a bridge tie. The tie between Ego 36 and Ego 50, i.e., the red edge line, indicates that resource flow in both directions, potentially connecting resources from Ego 36 to all the alters in Ego 50’s network (and vice-versa). Note that the network of Ego 59, one of Ego 36’s alters (top of the image, circled in blue) does not have bridge ties. Thus, the loss of any single member of this network would not result in the loss of food access to any other network members. Ego 36’s alter, Ego 59, is thus an example of an alter that is part of a dense network, of which Ego 36 is also a part, yet Ego 36 has potential access to unique, or non-redundant, food sources through its bridge ties to Ego 50 and Alt192 which are only available to Ego 59 through Ego 36.

Network quality is particularly important for understanding perceived food security in Indigenous and multiethnic settings. First, bridge‑tie connections can provide indirect access to diverse food sources that would otherwise be unavailable to an actor’s immediate subgroup. Second, occupying or accessing these strategic positions is associated with higher prestige and with reported perceptions of greater food security [22,27,40,72]. Thus, network quality reflects not simply the number of connections an actor has, but the strategic value of those connections within the broader community network. These patterns are associated with increased integration among subgroups in multiethnic communities and with specialization in food production and social cooperation [53,54,72–75].

Although prior research shows that food‑sharing networks support food access among rural Indigenous and agrarian populations, far less is known about how these relationships operate in peri‑urban, multiethnic Indigenous communities. Existing studies rarely integrate household‑level food insecurity measures with detailed egocentric network data, leaving open questions about how network size, density, and quality relate to perceived food security in such settings. To address this gap, we compare results from a locally adapted version of the Brazilian Food Insecurity Scale (*Escala Brasileira de Insegurança Alimentar –* EBIA), which measures household perceptions of food access, with egocentric food‑sharing network data to examine how households in Parque das Tribos are embedded within broader patterns of exchange and cooperation and how these patterns are associated with reported household food insecurity [46,76,77].

Using these quantitative metrics, we explore how social network characteristics shape perceptions of food security among Indigenous and agrarian households in peri‑urban settings. Larger networks can expand the number of potential types of food available to households, while denser networks may provide more stable and reliable support through tightly interconnected alters. At the same time, bridge‑tie connections, or links to alters who are not themselves directly interconnected, can offer access to more diverse food sources and are thus often associated with higher prestige and strategic positioning within community networks. Together, these patterns indicate that network size, density, and quality may each play distinct roles in shaping how households perceive their food security within contexts marked by irregular wage labor, conditional cash transfer (CCT) programs, and limited formal employment.

Specifically, we advance the following hypotheses:

1. Consistent with prior research showing that social networks support food access among Indigenous and rural populations, we expect households embedded in larger, denser, and higher‑quality egocentric food‑sharing networks to report lower levels of household food insecurity as measured by a locally adapted EBIA instrument.
2. Household demographic characteristics, economic conditions, and the multiplexity of food-related social interactions will be associated with variation in egocentric network size, density, and quality.
3. Households engaged in interethnic and interregional cooperation will exhibit larger, more interconnected, and higher‑quality egocentric networks, reflecting the role of cross‑group ties in shaping household embeddedness within the broader community.

## Methods

### Research setting

Parque das Tribos (EN: Tribes’ Park) is located on the westernmost periphery of the city of Manaus in the Brazilian Amazon, near the confluence of the Tarumã Açu River and the Rio Negro, an area long favored by Indigenous migrants [78,79]. According to the 2018 census, Parque das Tribos was home to 478 Indigenous, mixed Indigenous, non-Indigenous, and undeclared or unregistered Indigenous families, representing 34 distinct ethnicities [78,80]. In addition to Portuguese and the Amazonian *lingua franca* Nheengatu [81,82], the most-spoken languages within the community include Arawakan languages (Baniwa, Kuripako, Apurinã, and Kanamarí), Tikuna, Kokama, Sataré Mawé, and Tukanoan languages (Tukano, Dessana, Tuyuka, Wanano, and Piratapuyo) [78] (conversations with community leaders).

Most Indigenous residents of Parque das Tribos are migrants from one of the three major cultural/ecological regions within the state of Amazonas, the Upper Rio Negro, the Upper Solimões/Amazon River, and the Middle Amazon/Madeira Rivers. The non-Indigenous residents had varied origins, including the same cultural/ecological regions as the Indigenous residents, as well as other Brazilian states.

Our ethnographic research provided insights into residents’ strategies and challenges securing a livelihood and food. The neighborhood’s geographic location and a lack of urban planning make travel to Manaus’ city center for work or to access goods and services difficult [83]. Most residents rely on municipal buses with erratic schedules which make the 25 km trip an all-day commitment. As a result, livelihood strategies among residents of Parque das Tribos reflect the reality of most Indigenous residents of urban centers in the Brazilian Amazon, i.e., irregular wage labor [54], reliance on conditional cash transfer programs like the “Bolsa Família Program” (BFP) [58], and the sale of arts and crafts to tourists [84].

### Data collection

We collected data from September 12 – October 30, 2022, as part of a larger project on food security in Parque das Tribos. All research activities were approved by ethics committees in the USA and Brazil. In the USA, the protocol was approved by The Ohio State University Institutional Review Board (IRB) (Study No. 2021E0240). In Brazil, the study was originally approved at the federal level by the *Comissão Nacional de Ética em Pesquisa* – CONEP (National Commission for Ethics in Research) (Case No. 3.687.909) and later resubmitted as part of the project entitled *“C&T AGROFLORESTA: Uso de tecnologias para a redução do desmatamento e inclusão socioprodutiva de comunidades rurais em situação de vulnerabilidade socioeconômica”* (“S&T: The use of technologies for the reduction of deforestation and socio-productive inclusion of rural communities in situations of socio-economic vulnerability”), under the same coordination of Prof. Dr. Therezinha de Jesus Pinto Fraxe due to a delay in the project chronology resulting from the COVID-19 Pandemic (S1 File). All participants received orientation prior to any interviews on informed consent during which they could ask questions. Each potential participant was then given a copy of the informed consent form, which they signed after any questions/concerns were addressed. Data collection was conducted with the approval of the Parque das Tribos community leadership and in accordance with the community’s expectations for respectful research engagement. Participants provided informed consent, and all procedures complied with the ethical guidelines of the relevant institutional review boards. Consistent with principles of Indigenous data sovereignty, data were stored securely, shared only in aggregate form, and used exclusively for the purposes described to participants.

All network datasets were fully de‑identified prior to analysis (names, household identifiers, and any information that could reasonably identify individuals were removed). All nodes were represented using randomized alphanumeric codes, and all network visualizations were generated exclusively from these de‑identified datasets.

To recruit participants, the first author and local research assistants used census data and contact information for Parque das Tribos provided by community leaders. Residents with a telephone number registered with community leadership were contacted via WhatsApp. Others were recruited through orientation meetings held at the *Maloca dos Povos Indígenas* (Indigenous Peoples’ Longhouse) community center. Our final sample consists of 106 residents, each representing an individual household. Table 1 reports household demographic and economic data for the sample. Because recruitment relied on leader‑provided contact lists and community‑center meetings, our sample may overrepresent households already connected to local institutions, which could influence observed patterns of social connectivity. Women comprised 86% of respondents, reflecting gendered labor and knowledge around food provisioning [23,85]. Response and refusal rates were systematically recorded and are reported in a supplementary file (See S2 File) to clarify representativeness.

**Table 1:** Household demographic and economic data for the n = 106 participants in the egocentric network interviews.

Paricipant Data			
		N	Percent
Sex	M	15	14%
	F	91	86%
Age	18-24	11	10%
	25-34	22	21%
	35-44	20	19%
	45-54	22	21%
	55-64	22	21%
	65+	9	8%
	Average age	44.0 (±14.9)	
Time in Parque das Tribos	1-2yrs	24	23%
	3-4yrs	19	18%
	5-6yrs	25	24%
	7yrs	38	36%
Marital Status	Single/Unmarried	62	58%
	Married/Civil Union	35	33%
	Divorced/Separated	2	2%
	Widowed	7	7%
Household Data			
Household Composition	Oldest Household Member (average)	46.1(±14.6)	
	Household size (average)	3.9 (±1.9)	
	Number of Adults/Household	2.3 (±1.4)	
	Number of Children/Household	1.6 (±1.4)	
	Single Parent Household, Male	9	8%
	Single Parent Household, Female	15	14%
Sources of Income	No reported or informal income	26	25%
	Bolsa Família Program (BFP) only	39	37%
	Wage Earner in Household only	33	31%
	Bolsa Família Program (BFP) and Wage Earner	8	8%
Education Level	No formal education	9	8%
	Some primary school or complete	54	48%
	Some high school or complete	36	34%
	Some college and above	7	7%
Means of Self-Production	Garden	57	54%
	No Self-Production	29	27%
	Hunting, Fishing, and Foraging	28	26%
	Cabin	18	17%
	Domestic Animals	17	16%

The 106 participants in our study, as well as the 339 people whom they identified in social network interviews as exchange partners, represented four distinct cultural/geographic regions of the Brazilian Amazon: The Upper Rio Negro (URN), the Upper Amazon/Solimões (UAS), the Middle Amazon/Madeira (MA), and non-Indigenous/Not Certain/Undeclared (NSI). Study participants represent 28 of the 34 distinct ethnic groups within *Parque das Tribos* (Table 2).

**Table 2:** Regional and ethnic origins of egos and alters identified in the egocentric network interviews.

Region of Origin (Egos)			Region of Origin (Alters)		
Upper Rio Negro	34	32%	Upper Rio Negro	91	27%
Upper Amazon (Solimões)	28	26%	Upper Amazon (Solimões)	49	14%
Middle Amazon / Madeira River	25	24%	Middle Amazon / Madeira River	55	16%
Non-Indigenous/Not Certain/Undeclared	19	18%	Non-Indigenous/Not Certain/Undeclared	144	42%
Ethnicity (Egos)			Ethnicity (Alters)		
Mura	14	13%	Baré	37	11%
Tikuna	12	11%	Mura	32	9%
Baré	11	10%	Tikuna	21	6%
Kokama	8	8%	Tariano	20	6%
Munduruku	7	7%	Tukano	14	4%
Dessana	5	5%	Kokama	14	4%
Sataré Mawé	4	4%	Sataré Mawé	10	3%
Tariano	4	4%	Munduruku	9	3%
Tukano	4	4%	Baniwa	8	2%
Baniwa	3	3%	Dessana	5	2%
Canamari	3	3%	Miranha	4	1%
Miranha	2	2%	Kambeba	3	1%
Apurinã	2	2%	Warao	3	1%
Wanano	2	2%	Wanano	3	1%
Piratapuyo	1	1%	Apurinã	2	1%
Tuyuka	1	1%	Kaixana	2	1%
Kaixana	1	1%	Tuyuka	1	0.50%
Koripako	1	1%	Witoto	1	0.50%
Arapaço	1	1%	Koripako	1	0.50%
Macuxi	1	1%	Mirity-tapuya	1	0.50%
Non-indigenous/Not Certain/Undeclared	19	18%	Canamari	1	0.50%
			Macuxi	1	0.50%
			Morubo	1	0.50%
			Jê	1	0.50%
			Non-indigenous/Not Certain/Undeclared	144	43%

Once recruited, all participants were administered a 2-part survey at the community center. The first part included data on household demographics, economics, and a version of the Brazilian Food Security Scale (EBIA), which assesses perception of household food‑insecurity experiences over the previous 30 days [77,86], and specifically adapted for use in the community [87]. Minor wording adjustments were made to improve comprehension, but the structure and intent of each item remained consistent with the standardized EBIA. All questions were administered orally by trained research assistants in Portuguese, with clarification provided in Nheengatu when requested by participants. The EBIA includes 14 scored items for households with children, yielding a scale of 0–14 points, and 8 scored items for households without children, yielding a scale of 0–8 points. Because these raw scores are not directly comparable across household types, we used the standard EBIA categorical classifications – Food Secure (0 points), Light Food Insecurity (1 point), Moderate Food Insecurity (2 points), and Severe Food Insecurity (3 points) – based on the published scoring ranges for each version of the instrument [77,86]. These four categories were then used to create a single, comparable ordinal variable for all regression models (See S3 File).

The second part of the survey was an egocentric network interview. For these interviews, we applied 8 *name generator* questions. Specifically, we asked participants to list all the people (i.e., alters) with whom they or someone in their household had interacted over the previous six months in relation to food, money, hunting/fishing/foraging, and agricultural aid, emphasizing both the giving and receiving of support. Responses indicating more than one category per exchange partner signifies a *multiplex* relationship. After naming all individuals for each question, participants were asked to provide *name interpreters* [64,88,89], qualifying each alter with regards to the nature of their relationship (i.e., family, neighbor, colleague, acquaintance, etc.), their ethnicity, whether they lived in Parque das Tribos, the frequency of exchange (1 = one time in past six months, 2 = twice in past six months, 3 = three times or more in past six months), and the items given or received [64,89]. (See S4 File for the Egocentric network interview form). These data provide information about the relationships between participants and the network structure of the community. Because the EBIA assesses food‑insecurity experiences over the previous 30 days, whereas the network instrument captures food‑sharing interactions over the previous six months, the two measures reflect different but complementary time frames: recent household food‑insecurity experiences and longer‑term patterns of social exchange.

In the case of the latter, this is accomplished by linking the interviewed individual, or ego, through mutual alters to other egos through a process called “link tracing” [20,90,91]. Link tracing allows for identifying which egos and alters are more centralized, as well as calculating the overall network size, density, and quality for each ego within the overall network. We analyzed alter data from the name interpreters during each egocentric interview to identify those alters who were potentially shared by multiple egos. This provided us with a better understanding of the level of interconnectivity among the individual egocentric networks, as well as the density of the overall network. An understanding of the level of interconnectivity between the individual egocentric networks provides insight into the level of interethnic cooperation within the community [54,55,92–94]. We then conducted subsequent interviews with those egos, as well as consulting with community leaders, to verify whether the alters were indeed the same for each respective ego. Finally, we calculated the network size, density, and quality of each ego as part of the overall network.

### Data analysis

#### Hypothesis 1: Network structure and perceived food security.

To evaluate H1, which predicts that egocentric network characteristics (size, density, and quality) are associated with perceived household food security, we modeled EBIA category as an ordinal outcome using hierarchical Bayesian regression with a Gaussian likelihood. Network size, density, and eigenvector‑based network quality were each scaled prior to analysis. Models were estimated both with and without household size, given prior evidence that larger households report lower perceived food security. All models were implemented in the rstanarm package in RStudio [95,96] using four MCMC chains of 1,000 iterations each and weakly informative normal priors [27,97–99]. Variance inflation factors (VIFs) were calculated to assess multicollinearity among predictors. These models directly test whether the three network measures identified in the literature as central to food‑sharing strategies predict EBIA category.

#### Hypothesis 2: Demographic, economic, and multiplexity variables → network structure.

To evaluate H2, which predicts that household demographic characteristics, economic conditions, and the multiplexity of food‑related social interactions shape egocentric network structure, we modeled each network measure (size, density, and quality) as a continuous outcome in separate hierarchical Bayesian regressions. Predictors included household composition (e.g., number of older household members, single female head of household), economic indicators (formal employment, autonomous employment, cash‑transfer participation, monthly expenses), and behavioral measures of social exchange (multiplexity, inward and outward sharing ties). Each model used a Gaussian likelihood with weakly informative priors and was estimated using the same MCMC structure described above. VIFs were calculated for each regression equation. These models test whether demographic and economic constraints, together with multiplexity, contribute to variation in network size, density, and quality.

#### Hypothesis 3: Interethnic and interregional cooperation → network embeddedness.

To evaluate H3, which predicts that interethnic and interregional cooperation contribute to household embeddedness within the broader community network, we modeled network quality, density, and size as outcomes in Bayesian regressions including measures of cross‑group interaction. Predictors included regional homophily, proportion of alters from Parque das Tribos, and indicators of interethnic or interregional cooperation. These models assess whether households engaged in cross‑group exchange exhibit larger, more interconnected, and higher‑quality networks. As above, models were estimated using rstanarm with Gaussian likelihoods, weakly informative priors, and four MCMC chains of 1,000 iterations each. All models were estimated using Markov chain Monte Carlo sampling with four chains of 1,000 iterations each, employing weakly informative normal priors.

#### General procedures.

All analyses were conducted in R using the igraph, ggraph, car, bayestestR, rstanarm, moments, and insight packages. Credible intervals were set at 89%, following recommendations for Bayesian interval interpretation. We report 89% credible intervals following contemporary Bayesian conventions emphasizing estimation over threshold‑based significance testing, consistent with recommendations by McElreath [100] and Makowski et al. [97]. Full model code is provided in S9 File. Weakly informative priors were used throughout due to limited prior evidence for large effects in similar studies, allowing estimates to remain agnostic in direction while constraining extreme values unless supported by the data.

## Results

### Hypothesis 1: Network structure and perceived food security

Of the 106 households, 25 (23.5%) were food secure or experiencing light food insecurity, 44 (41.5%) were moderately food insecure, and 37 (35%) were severely food insecure. Across the 106 egocentric food‑sharing networks, egos had a mean of 4.7 (SD = 2.7) alters, with an average of 3.8 (SD = 2.2) inward sharing ties and 3.8 (SD = 3.4) outward sharing ties. When weighted by frequency, egos had an average of 9.0 (SD = 6.3) inward sharing ties and 9.5 (SD = 10.1) outward sharing ties. Inward and outward ties were moderately correlated (r = 0.46), indicating that individuals who reported more outward sharing also tended to report more inward sharing. The proportion of reciprocated ties was 48.7%, providing a descriptive indicator of mutual exchange without modeling actor‑ or dyadic‑level dependencies.

Fig 2 presents the proxy network constructed from the 106 egocentric networks using link tracing to identify common alters.

**Fig 2:**
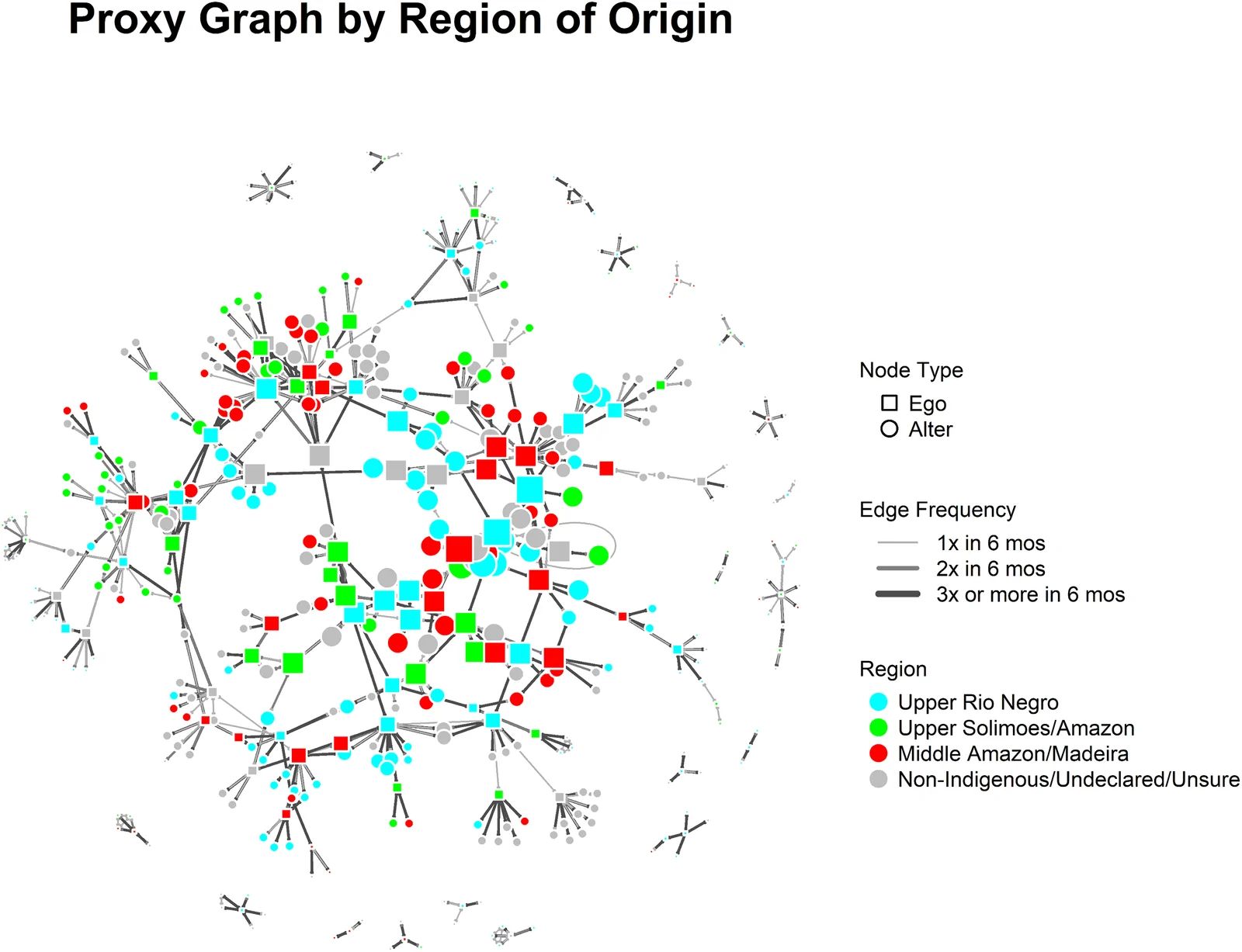
Proxy graph of the community food sharing network. Link tracing was used to identify common alters across the 106 egocentric networks, producing a proxy representation of the broader community network. Larger nodes indicate higher network quality (eigenvector centrality). Colors represent region of origin; the absence of color clustering indicates minimal regional homophily, suggesting that food‑sharing networks regularly include individuals from different regions of origin.

Analysis of the structural characteristics of network size, quality, and density indicates that all three have a probable association with positive perception of food security. As network size, quality, and density increase, the probability of being more food secure also increases. Inclusion of household size has a clear negative effect on perception of food security but does not alter the predictive effects of the network variables. Fig 3a–3b show the predicted effects of the network measures on perception of food security with and without household size included. Table 3 reports the posterior medians, credible intervals, probabilities of direction, and VIFs.

**Table 3:** Posterior Median Effects, Credible Intervals (CI), Probabilities of Direction (PD), and Variance Inflation Factors (VIF) for the network variables of interest. Household Size was included only in the first model; therefore, estimates are omitted (“x”) in the second specification.

Predictor	Posterior Median (w/ Household Size)	89% Credible Interval (w/ Household Size)	Probability of Direction (PD) (w/ Household Size)	Posterior Median (w/o Household Size)	89% Credible Interval (w/o Household Size)	Probability of Direction (PD) (w/o Household Size)
Network Size	0.19	[0.04, 0.34]	98.3%	0.18	[0.03, 0.33]	97.3%
Network Density	0.09	[0.04, 0.33]	97.9%	0.20	[0.06, 0.35]	99.0%
Network Quality	0.13	[0.01, 0.25]	96.0%	0.13	[0.02, 0.25]	96.3%
Household Size	−0.18	[-0.30, -0.07]	99.3%	x	x	x
VIF (w/ Household Size)				VIF (w/o Household Size)		
Network Size	Network Density	Network Quality	Household Size	Network Size	Network Density	Network Quality
1.53	1.53	1.00	1.01	1.52	1.51	1.01

**Fig 3:**
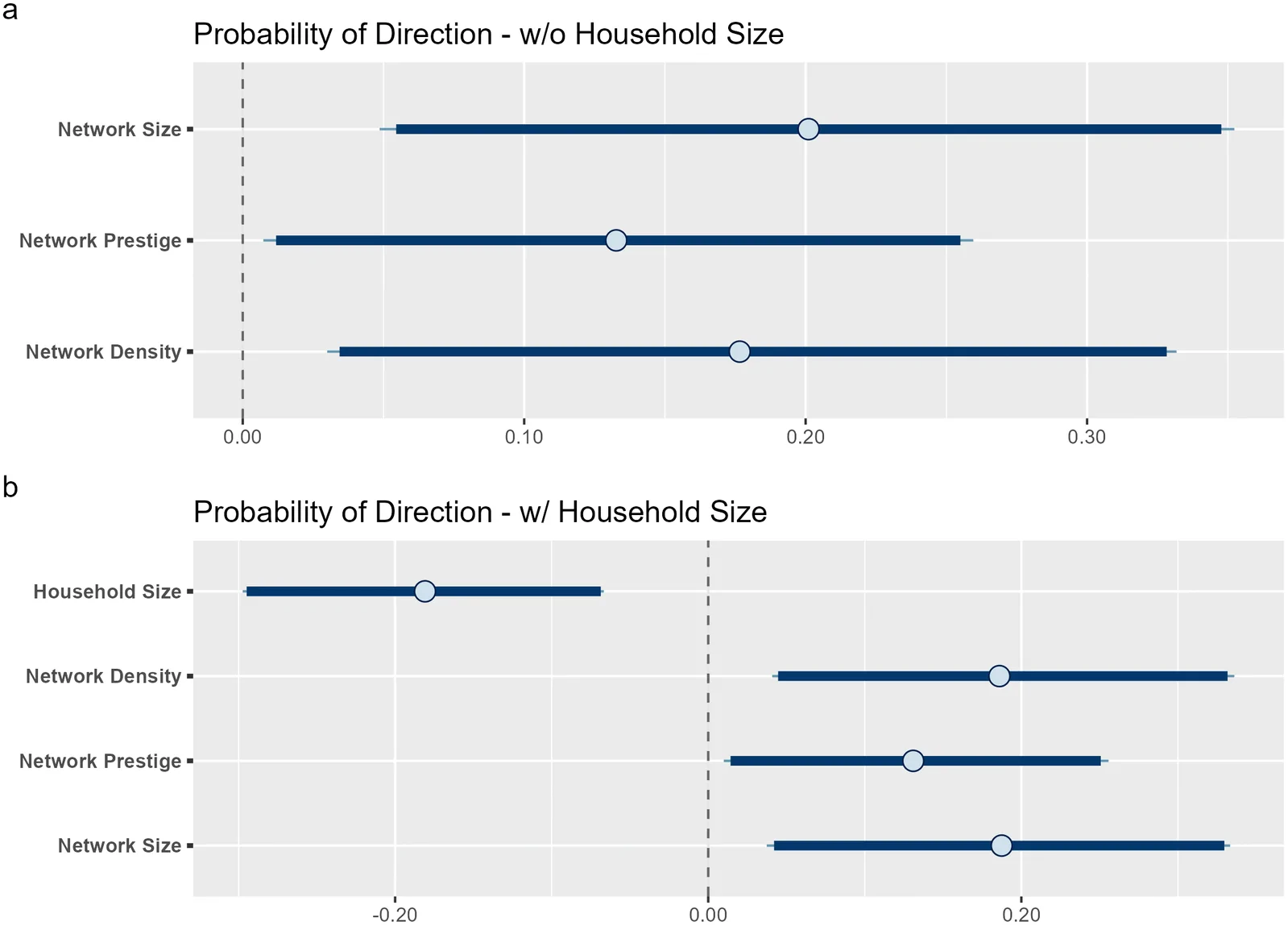
Effects of key network measures on perception of food security, measured against household economic variables. Fig 3a (left) shows these same network measures with the variable Household Size added. Household Size is commonly associated with food insecurity. Greater network size, quality, and density are positively associated with higher reported food security, even among larger households. Fig 3b (right) shows the network measures of Size, Quality, and Density, measured against household economic variables, and their respective predictive effects on the overall perception of food security, as measured by the Brazilian Scale of Food Insecurity (EBIA) survey Instrument.).

### Hypothesis 2: Demographic, economic, and multiplexity variables and network structure

Regression results for network size, quality, and density are presented in Fig 4a–4c. These models examine the demographic, economic, and network variables of interest that may influence the structural characteristics of food‑sharing networks.

**Fig 4:**
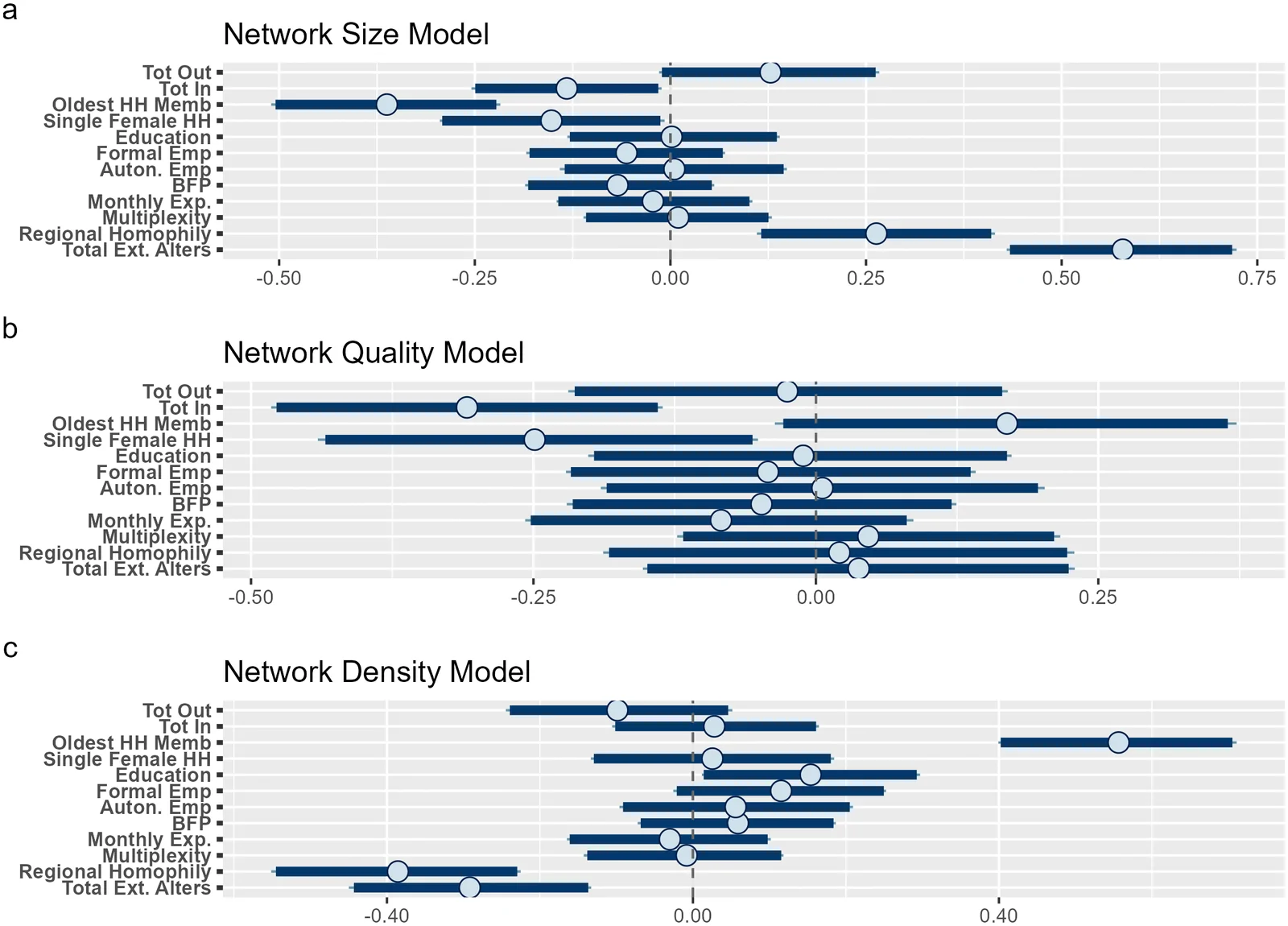
Posterior distributions of demographic, economic, and network variables predicting network size (a), quality (b), and density (c). Thick lines represent 50% credible intervals; thin lines represent 89% credible intervals. Numeric variables were scaled with normal distributions.

Multiplexity, referring to multiple categories of food‑related support, has a clear negative effect on network size, a slight positive effect on network density, and a clear positive effect on network quality. Monthly expenses have a negative effect on both network size and quality, while informal day work and receiving BFP have minimal positive effects on network density.

Regional homophily, or having a network comprised of households originating from the same cultural or geographic region, has a slight negative effect on network size and a clear negative effect on network quality. Less regionally homophilous networks result in greater overall network quality, as indicated by ego’s prestige. Fig 2 illustrates this pattern in the proxy network. Full details of prior and posterior distributions are provided in S4 Table.

### Hypothesis 3: Interethnic and interregional cooperation and network embeddedness

To illustrate how cross‑group cooperation relates to network embeddedness, Fig 5a–5b present two egocentric networks at two degrees (ego – alters – alters’ alters). These examples visually demonstrate how variation in network size, density, and quality corresponds to differences in perceived food security. Full details of variables of interest are provided in S4 Table and S8 File.

**Fig 5:**
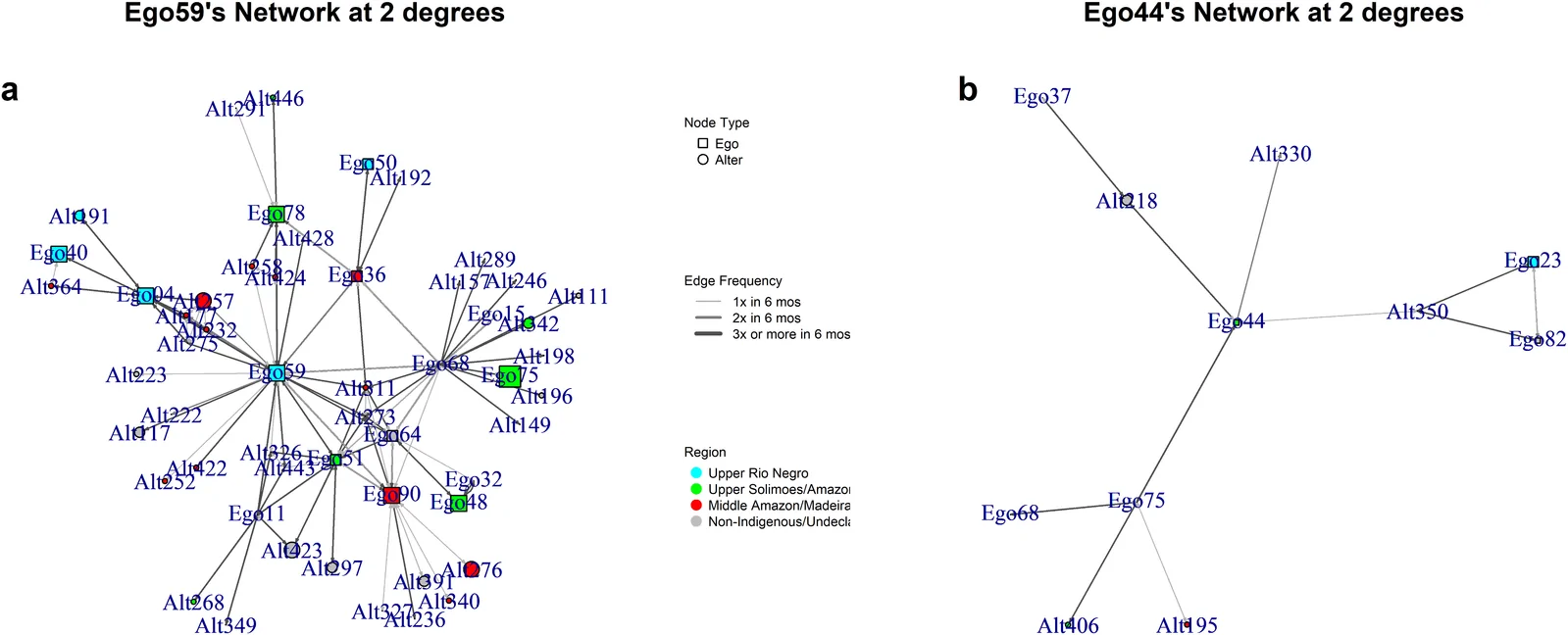
Individual networks at two degrees. Fig 5a shows an ego experiencing mild food insecurity whose network size, density, and quality all fall within the upper quartile (≥75%) of the community. Fig 5b shows an ego experiencing severe food insecurity whose network measures fall below the sample median (≤50%). Larger nodes indicate higher network quality. Fig 5a. Example of a larger, denser network. Ego59, mild food insecurity, has no wage‑earning household members, receives BFP, has a household size of 4 (including 3 children), and network size, density, and quality in the upper quartile (>75%), demonstrating the predictive effect of denser, larger, higher‑quality networks on perception of food security. Fig 5b. Example of a smaller, less dense network. Ego44, severely food insecure, is formally employed, receives BFP, has a household size of 5 (including 1 child), and network size, density, and quality below the respective medians (<50%). The lack of larger vertices indicates relatively low prestige, and the limited interconnectivity among alters reflects lower network density.

## Discussion

The aim of this study was to determine how social capital, as measured by the social network variables of network size, quality, and density, influences perceptions of household food security in the multi-ethnic, predominantly Indigenous peri-urban Amazonian community of Parque das Tribos. Our findings indicate a clear positive association between participants’ social capital and their perception of food security. This confirms the findings of previous research conducted in rural and peri-urban settings [22,27,47,51,101,102]. Furthermore, our results suggest that higher social capital is positively associated with reported food security even among larger households, where financial resources may be insufficient. This is important, as other studies have found a negative association between household size and perceived food security in rural and urban settings, presumably due to anxiety over having more individuals to feed [15,29,103–105].

In this sample, the three social network measures – size, density, and quality – were more consistently associated with reported household food security than the economic indicators we included. Nevertheless, we caution that this pattern should not be interpreted as evidence that economic capital is unimportant for food access in peri-urban Indigenous communities; rather, it reflects the specific conditions of Parque das Tribos, where irregular wage labor, unreliable public transport, limited formal employment, and reliance on conditional cash transfer programs shape how households experience and report food security. In this context, social capital, as characterized by the network variables, appears to function as a salient dimension of household food access, while the economic variables measured here showed weaker associations. It is important to note that the EBIA captures reported household experiences of food insecurity rather than dietary quality, nutrient adequacy, or anthropometric status, and our interpretation reflects this distinction [20,76,87]. Thus, these findings align with prior research demonstrating that social exchange networks can remain central to food access in mixed-economy Indigenous settings, even where money-based markets are present. However, the associations observed here should be understood as context-specific and cross-sectional, rather than indicative of a more generalized contrast between social and economic capital.

Residents navigate a mixed‑economy environment shaped by irregular wage labor, limited access to formal employment, and reliance on conditional cash‑transfer programs, all of which constrain the stability of monetary resources available for food acquisition [23,58,87]. These structural conditions contribute to the continued importance of social exchange networks, as households rely on community ties to buffer short‑term gaps in food access and to manage the unpredictability of urban labor markets. At the same time, money‑based markets, municipal services, and diverse livelihood strategies mean that social capital operates alongside, rather than in place of, economic capital. Thus, the associations documented here reflect the interaction between structural precarity in the money‑based economy and longstanding practices of reciprocity‑based cooperation, rather than a simple substitution of social capital for economic resources.

### Examining the structural characteristics of networks

Our findings demonstrate that all three network measures – *size, quality,* and *density* – are positively associated with improved perception of food security. Yet the manner in which they function in this setting differs from previous studies in rural settings [22,45,106]. Network size and density are both positively associated with household food security, yet they are also partly connected [27], with larger networks tending to be less dense. Thus, when looking at network density, we had to control for network size to determine which factors, if any, affected density besides network size. Our results showed that multiplexity, while resulting in greater network density, also resulted in lower network size (Fig 3a–3c). When standing alone, or individually with network quality, the probable effect is considerably lessened, although network quality alone maintains its positive predictive effect on perception of food security. This is not surprising; in the context of food sharing in our study, this is characterized by the correlation between inward and outward sharing ties, in which an increase in outward support results in a similar increase in inward support. In other words, the more people to whom an individual provides food or other forms of food assistance, the more that person will receive in return, with a greater variety of items becoming accessible as the number of partners increases. This, in turn, contributes to higher prestige for the person within the network, as they not only become a potentially unique source of varied food items by being a bridge between subgroups, but also play an important central role in the whole network [67,107,108]. This confirms the findings of previous studies among Indigenous and rural groups, including in the Amazon, in which one’s prestige or social standing is associated with access to varied or unique items [22,27,109].

On the other hand, the fact that network size and density together have a positive effect on perception of food security, whereas neither has nearly the same effect when measured alone, may seem surprising. It should not be. The correlation between network size and density has been well documented [22,27,65,110]; although in many cases as network size increases density decreases [22,27,106]. In contrast, our findings align with Burt [65], who proposes that social capital, manifested by positions of advantage within a network such as those occupied by actors in bridge ties between dense subgroups, can result in overall high network size and density. These highly prestigious connections can thus introduce greater variety of food products even in the densest subgroups while maintaining the consistency of strong ties associated with dense networks [22,27,111]. Our findings in this regard are not unique; in fact, such social network structures have long existed in urban centers among ethnic and religious minorities who, for reasons of homophily or marginalization, developed social systems with strong foundations in mutual support, such as in historical Jewish quarters and urban “ghettos” [112–115], as well as Amazonian rural and Indigenous migrant communities [50,54].

### Economic vs. social capital

Unlike previous studies, our data do not indicate a clear association between households having sources of income and perception of food security (Fig 4a–4b) [28,116,117]. In fact, an important and counter-intuitive finding is that households with a formally employed family member demonstrated a greater likelihood of reporting EBIA scores which indicate more severe food insecurity (41%) than those without a formally employed member (31%). One explanation is the high level of inflation in Brazil, which directly affects food prices. Another is that this study was conducted just after the last large wave of the COVID-19 pandemic and associated public health quarantines. During this time, many employed residents had their income interrupted, resulting in uncertainty regarding the ability to rely on wages to purchase food [118–120].

Beyond economic considerations, psychological considerations could present an important factor. Social support has been shown to serve as a buffer against adverse conditions (“buffering model”) in addition to being a tool for assessment of an individual’s degree of integration within a larger social network (“main effect model”), both of which improve mental well-being [121]. Our data support this. In this case, there is a resulting feeling of security with regard to food access through a sense of belonging to a group, as reflected in network size, quality, and density. In this setting, food sharing as a means of access appears primarily as a social phenomenon based on reciprocity between actors [122,123], although it intersects with economic resources in complex ways. In contrast, obtaining food by means that are transactional, including purchasing food via monetary means, does not necessarily result in similar mental well-being attributed to social support. Consequently, there is a distinct uncertainty regarding one’s ability to depend on monetary means of access to food, despite the majority (97%) of participants indicating that food expenditures were a regularly occurring household expense. Thus, while social capital, as measured by *network size, quality,* and *density,* is positively associated with reported household food security in this sample, comparable associations with economic capital were not observed in our models.

In this setting, wage earning was associated with greater household expenses, but also with a lower likelihood of engaging in social networks, i.e., we found a negative association between monthly expenses and network quality (Fig 3b). In contrast, the positive association between receipt of funds from the BFP and predicted network density warrants further investigation into the specific items exchanged in these networks. For example, BFP-recipient households might exchange cash-purchased food and non-food items for food items obtained by other means including hunting, fishing, foraging, or gardening. Such exchanges have been documented in rural Amazonian communities [61,124,125].

Our findings reinforce those of prior studies showing that greater involvement in the cash economy is associated with lower overall network quality and greater network isolation [23,27,126,127]. Furthermore, our findings indicate that egocentric food-sharing networks in peri-urban and urban settings like the ones observed in Parque das Tribos frequently serve the same social support functions that exist in food-sharing networks in rural areas, despite the presence of cash-based markets [25,45,46,61,128,129]. In this way, our data indicate that, at least in this setting, regularly accessing food via social networks is positively associated with higher reported food security, even when there is no interruption to other means of food access (e.g., cash). This may be related to the important role of network participation in generating trust and community identity, both among Amazonian Indigenous and riverine populations and Indigenous populations elsewhere in the Americas [28,35,41,51,130]. Furthermore, in this social setting, it could explain why individuals who lack regular social network integration may perceive themselves as food insecure, even when earning a regular income and having access to market-based food sources.

### Interethnic cooperation

Finally, our findings confirm those of previous studies on large-scale Indigenous interethnic cooperation in peri-urban and urban settings [22,39,51,55,131,132]. For example, we found higher overall network size and prestige associated with greater interethnic and interregional cooperation (see Fig 2). This is not surprising; urban and peri-urban Indigenous and rural populations both in Brazil and elsewhere have long demonstrated a propensity for interethnic or “supra-ethnic” cooperation, including among individuals from different cultural or geographic origins, particularly in settings where they face marginalization or outright discrimination [50,54,55,93,94,133–136]. We demonstrate a clear example of the “urban Indigenous” phenomenon, characterized by a dual identity based simultaneously on common indigeneity yet preservation of individual ethnicity, as described in the ethnographic literature both within Brazil and elsewhere [54,55,93,94,137–139]. Furthermore, we have provided here quantitative data of this interregional and interethnic cooperation in a peri-urban setting, in which reciprocal food sharing is closely associated with positive perception of food security. Qualitative responses during the network interviews indicate that the resources circulating through these ties were ecologically diverse. Participants described exchanges involving prepared meals and staple ingredients but also items obtained through hunting, fishing, and small-scale foraging, as well as garden produce such as bananas, *macaxeira* (a variety of manioc), herbs used in teas, and *cheiro verde* (a common mix of parsley and scallions). These mixed flows of purchased and self-produced foods could potentially help explain why actors occupying bridge tie positions reported higher network quality: They connect households to resource types that differ in origin, seasonality, and reliability. This confirms previous findings in both rural and peri-urban/urban settings that question the applicability of standardized food security survey instruments that focus primarily, or even entirely, upon access to food sources by monetary means [46,76,87,140].

In rural settings, these reciprocal relationships are often rooted in hierarchical structures carried over generations [30,111,141,142] and serve as a primary means of food access. However, despite the presence of other individuals of similar ethnic or geographic origins, these traditional relationships do not appear to dominate food sharing in the case of peri-urban and urban settings. Rather, as our study clearly demonstrates, reciprocal food-sharing regularly crosses interethnic and interregional lines and is associated with greater overall stability within the networks and more regular access to varied food sources, even in times of hardship. As one participant explained, “We give chicken eggs to ‘parentes’ and they give us fish, bananas, manioc flour, you know.” Given the well-documented importance of reciprocal relationships in both rural and Indigenous Amazonian identity and community integration that frequently crosses ethnic and linguistic boundaries [31,35,134,143,144], sharing thus becomes a social, rather than a purely economic system [27,31,51,54,92,145,146].

## Conclusion

In peri-urban areas where rural traditions intersect with urban market forces, food-sharing networks appear to serve as a critical buffer against food insecurity associated with economic uncertainty. Our findings support previous research findings establishing a relationship between participation in food-sharing networks and perceived food security. Importantly, the minimal, yet positive, impact of participation in the Bolsa Família conditional cash transfer program on network density suggests that government financial assistance may complement, rather than replace, food-sharing networks as a means of food access. In contrast, the absence of a link between wage earning and food-sharing network participation raises important questions about the structural limitations of income through labor as a pathway to food security in these areas. Further research is thus warranted to explore the respective roles of food-sharing relationships, conditional cash transfer programs, and market integration in peri-urban settings. Limitations

We acknowledge that our project, with its focus on egocentric networks, only partially reflects the organization, composition, and functionality of food-sharing networks within Parque das Tribos, particularly with regards to alter-alter relationships, which were based solely on information from egos. As such, our findings should serve as a basis for further, long-term research within the community, including determining more precisely the frequencies, types, and quantities of foods shared, the nature of other forms of food-related support, and the manner in which ego-alter relationships are initially established.

## Supporting information

S1 File Brazilian Ethics Approval. Copy of the Plataforma Brasil ethics approval and corresponding clarification.
(PDF)

S2 File Recruitment and Withdrawal Report. Table showing all recruited codes, including those who withdrew, as well as limited demographic information and means of recruitment.
(PDF)

S3 File Brazilian Food Insecurity Scale (locally adapted). The locally adapted food security survey instrument used in the community.
(PDF)

S4 File Egocentric Network Interview form. The questionnaires applied in the semi-structured interviews.
(PDF)

S5 Table Results of Statistical Analyses. Excel tables of the statistical analyses.
(XLSX)

S6 Fig Graphs of Prior and Posterior Distributions. Graphics of the prior and posterior distributions calculated in R.
(TIF)

S7 File Graphs of Socioeconomic Variables and Perception of Food Security. Two alternative graphs demonstrating the full network and control variable posterior distributions.
(PDF)

S8 Table Description of the Dataset. Full table of the dataset.
(XLSX)

S9 File R Studio code. Text file of the code used in calculations.
(TXT)

S10 File Inclusivity Questionnaire. (PDF)
