# Supplementary material for: Food sharing networks in a multi-ethnic, predominantly indigenous peri-urban community in the Brazilian Amazon
Source: PLoS One. 2026 Nov 11;21(11):e0360629. doi: 10.1371/journal.pone.0360629 (PMC13754111; doi:10.1371/journal.pone.0360629)
Supplement: Supplementary file 1 [file pone.0360629.s001.pdf]

DETALHAR PROJETO DE PESQUISA

DADOS DO PROJETO DE PESQUISA

Título Público: C&T AGROFLORESTA: Uso de tecnologias para a redução do desmatamento e inclusão socioprodutiva de comunidades rurais em situação de vulnerabilidade socioeconômica
Pesquisador Responsável: Therezinha de Jesus Pinto Fraxe
Contato Público: Therezinha de Jesus Pinto Fraxe
Condições de saúde ou problemas estudados:
Descritores CID - Gerais:
Descritores CID - Específicos:
Descritores CID - da Intervenção:
Data de Aprovação Ética do CEP/CONEP: 31/08/2022

PLATAFORMA BRASIL COORDENADOR PLATAFORMA BRASIL

DADOS DA INSTITUIÇÃO PROPONENTE

Nome da Instituição: Universidade Federal do Amazonas - UFAM
Cidade: MANAUS

DADOS DO COMITÊ DE ÉTICA EM PESQUISA

Comitê de Ética Responsável: 5020 - Universidade Federal do Amazonas - UFAM
Endereço: Rua Teresina, 4950
Telefone: (92)3305-4000
E-mail: cep@ufam.edu.br

CENTRO(S) PARTICIPANTE(S) DO PROJETO DE PESQUISA

CENTRO(S) COPARTICIPANTE(S) DO PROJETO DE PESQUISA

Voltar
