# Supplementary material for: Food sharing networks in a multi-ethnic, predominantly indigenous peri-urban community in the Brazilian Amazon
Source: PLoS One. 2026 Nov 11;21(11):e0360629. doi: 10.1371/journal.pone.0360629 (PMC13754111; doi:10.1371/journal.pone.0360629)
Supplement: Supplementary file 2 [file pone.0360629.s002.pdf]

	Ego Code	Code	Means of Recruitment	Gender	Age	Primary Daily Language	Fluent in Portuguese?
1	Ego39	4MF1-01	Maloca	M	F	4	Y
2	Ego25	5FG1-02	Maloca	F	G	3	Y
3	Ego43	1MF1-03	Maloca	M	F	1	Y
4	Withdrawn	6FF3-04	Maloca	F	F	1	Y
5	Withdrawn	1FF2-05	Maloca	F	F	1	Y
6	Withdrawn	7FE1-06	Maloca	F	E	5	Y
7	Withdrawn	4FE1-09	Maloca	F	E	1	Y
8	Ego100	9FG1-10	Maloca	F	G	5	Y
9	Ego70	7FG6-11	Maloca	F	G	5	Y
10	Withdrawn	1FC3-12	Maloca	F	C	1	Y
11	Withdrawn	1FD3-13	Maloca	F	D	1	Y
12	Withdrawn	10FE3-14	Maloca	F	E	1	Y
13	Withdrawn	5FD2-15	Maloca	F	D	3	Y
14	Ego08	6FDX-16	Maloca	F	D	7	Y
15	Withdrawn	10FD3-17	Maloca	F	D	1	Y
16	Ego33	6FD3-18	Maloca	F	D	7	Y
17	Withdrawn	11FD3-19	Maloca	F	D	7	Y
18	Ego26	6FD3-20	Maloca	F	D	7	Y
19	Withdrawn	6FC3-21	Maloca	F	C	7	Y
20	Withdrawn	5FE3-22	Maloca	F	E	3	Y
21	Withdrawn	12FE3-23	Maloca	F	E	1	Y
22	Withdrawn	8FE3-24	Maloca	F	E	1	Y
23	Withdrawn	5FE2-25	Maloca	F	E	1	Y
24	Withdrawn	7FH5-26	Maloca	F	H	1	Y
25	Withdrawn	13FXX-27	Maloca	F	E	1	Y
26	Ego09	14MI6-28	Maloca	M	I	1	Y
27	Withdrawn	3FG3-29	Maloca	F	G	1	Y
28	Withdrawn	10FC3-30	Maloca	F	C	1	Y
29	Withdrawn	15FE1-31	Maloca	F	E	1	Y
30	Withdrawn	16MF2-32	Maloca	M	F	2	Y
31	Ego106	8ME3-33	Maloca	M	E	1	Y
32	Withdrawn	14MF2-34	Maloca	M	F	1	Y
33	Ego85	8MG1-35	Maloca	M	G	1	Y
34	Withdrawn	8FE1-36	Maloca	F	E	1	Y
35	Withdrawn	8FE3-37	Maloca	F	E	1	Y
36	Withdrawn	15ME3-38	Maloca	M	E	8	Y
37	Ego89	2FE3-39	Maloca	F	E	1	Y
38	Withdrawn	2FD3-40	Maloca	F	D	1	Y
39	Withdrawn	8FE1-41	Maloca	F	E	1	Y
40	Withdrawn	8FD3-42	Maloca	F	D	1	Y
41	Withdrawn	8FD3-43	Maloca	F	D	1	Y
42	Withdrawn	8FC1-44	Maloca	F	C	1	Y

43	Withdrawn	8MD1-45	Maloca	M	D	1	Y
44	Withdrawn	3FG3-46	Maloca	F	G	1	1
45	Withdrawn	3MG1-47	Maloca	M	G	2	Y
46	Ego72	10FE1-48	Maloca	F	E	1	Y
47	Ego75	12FG1-49	Maloca	F	G	1	Y
48	Withdrawn	12FG3-50	Maloca	F	F	1	Y
49	Ego50	3FB3-51	Maloca	F	B	3	Y
50	Ego03	7FF3-52	Maloca	F	F	5	Y
51	Ego77	17FF1-53	Maloca	F	F	1	Y
52	Withdrawn	12MF3-54	Maloca	M	F	1	Y
53	Withdrawn	13FF3-55	Maloca	F	F	9	Y
54	Withdrawn	10MF2-56	Maloca	M	F	2	Y
55	Ego101	10MD3-57	Maloca	M	D	1	Y
56	Ego41	2FG3-58	Maloca	F	G	1	Y
57	Ego71	10FD3-59	Maloca	F	D	1	Y
58	Ego49	6MF1-60	Maloca	M	F	5	Y
59	Withdrawn	7MG1-61	Maloca	M	G	5	Y
60	Ego57	1FF3-62	Maloca	F	F	1	Y
61	Withdrawn	8FC3-63	Maloca	F	C	1	Y
62	Withdrawn	13FC2-64	Maloca	F	C	1	Y
63	Ego90	12FF3-65	Maloca	F	F	1	Y
64	Withdrawn	17FD3-66	Online	F	D	1	Y
65	Withdrawn	10FF3-67	Maloca	F	F	1	Y
66	Ego10	12FD3-68	Maloca	F	D	1	Y
67	Ego78	12FE3-69	Maloca	F	E	1	Y
68	Withdrawn	17FE3-70	Maloca	F	E	1	Y
69	Ego38	18FG3-71	Maloca	F	G	1	Y
70	Ego44	13FG5-72	Maloca	F	G	1	Y
71	Ego80	13FG1-73	Maloca	F	G	1	Y
72	Ego68	2FG3-74	Maloca	F	G	1	Y
73	Withdrawn	8FF3-75	Maloca	F	F	1	Y
74	Withdrawn	8FD1-76	Maloca	F	D	1	Y
75	Ego81	10FF3-77	Maloca	F	F	1	Y
76	Withdrawn	8FF1-78	Maloca	F	F	1	Y
77	Withdrawn	8FD1-79	Maloca	F	D	1	Y
78	Ego96	19FD3-80	Online	F	D	1	Y
79	Withdrawn	3FCX-81	Online	F	C	3	Y
80	Withdrawn	17FDX-82	Maloca	F	D		Y
81	Withdrawn	6FDX-83	Online	F	D	7	Y
82	Withdrawn	3FDX-84	Online	F	D	3	Y
83	Ego17	5FGX-85	Online	F	G	10	Y
84	Withdrawn	12MFX-86	Online	M	F	1	Y
85	Ego37	12FFX-87	Online	F	F	1	Y
86	Ego54	20FCX-88	Online	F	C	11	

87	Ego20	10FDX-89	Online	F	D	2	Y
88	Withdrawn	21FEX-90	Online	F	E	1	Y
89	Ego04	12FXX-91	Online	F	X	1	Y
90	Withdrawn	6MDX-92	Online	M	D	7	Y
91	Withdrawn	2FEX-97	Online	F	E	1	Y
92	Withdrawn	12FEX-95	Online	F	E	1	Y
93	Ego59	12FEX-96	Online	F	E	1	Y
94	Withdrawn	21FEX-97	Online	F	E	1	Y
95	Withdrawn	9MDX-98	Online	M	D	5	Y
96	Withdrawn	8FEX-99	Online	F	E	1	Y
97	Ego36	12ME3-100	Maloca	M	E	1	Y
98	Withdrawn	4ME1-101	Maloca	M	E	5	Y
99	Ego84	8FG3-102	Maloca	F	G	1	Y
100	Withdrawn	7FG3-103	Maloca	F	G	1	Y
101	Withdrawn	7MF3-104	Maloca	M	F	1	Y
102	Ego99	7FC3-105	Maloca	F	C	5	Y
103	Withdrawn	8FC2-106	Maloca	F	C	1	Y
104	Withdrawn	16FC3-107	Maloca	F	C	1	Y
105	Withdrawn	10FE3-108	Maloca	F	E	1	Y
106	Withdrawn	10FD3-109	Maloca	F	D	1	Y
107	Withdrawn	14MF2-110	Maloca	M	F	1	y
108	Withdrawn	4FF3-111	Maloca	F	F	5	Y
109	Withdrawn	10MD3-112	Maloca	M	D	1	Y
110	Withdrawn	6FD2-113	Maloca	F	D	7	Y
111	Withdrawn	10FE1-114	Maloca	F	E	1	Y
112	Withdrawn	10FF2-115	Maloca	F	F	1	Y
113	Withdrawn	8FD3-116	Maloca	F	D	1	Y
114	Withdrawn	8FE3-117	Maloca	F	E	1	Y
115	Ego34	3FF5-118	Maloca	F	F	5	Y
116	Ego45	14FE3-119	Maloca	F	E	1	Y
117	Ego94	25FE1-120	Maloca	F	E	1	Y
118	Ego93	24FXX-121	Maloca	F	F	10	Y
119	Withdrawn	6FXX-122	Maloca	F		7	Y
120	Ego52	13FE3-123	Maloca	F	E	1	Y
121	Ego86	6FC1-124	Maloca	F	C	7	Y
122	Ego87	13FD3-125	Maloca	F	D	1	Y
123	Ego19	8FF1-126	Maloca	F	F	1	Y
124	Ego48	1FD3-127	Maloca	F	D	1	Y
125	Ego53	5FD3-128	Maloca	F	D	1	Y
126	Ego129	13MG3-129	Maloca	M	G	1	Y
127	Ego14	13FF3-130	Maloca	F	F	1	Y
128	Ego73	1FG3-131	Maloca	F	G	1	Y
129	Ego27	2FF1-132	Maloca	F	F	1	Y
130	Withdrawn	12FC3-133	Maloca	F	C	1	Y

131	Ego05	8FC1-134	Maloca	F	C	1	Y
132	Ego65	12FG3-135	Maloca	F	G	1	Y
133	Ego35	6FF1-136	Maloca	F	F	1	Y
134	Ego104	1FC3-137	Maloca	F	C	1	Y
135	Ego11	6FC3-138	Maloca	F	C	1	Y
136	Ego46	7MH5-139	Maloca	M	H	5	Y
137	Ego13	1FE3-140	Maloca	F	E	1	Y
138	Ego64	10FF3-141	Maloca	F	F	1	Y
139	Ego32	12FD3-142	Maloca	F	D	1	Y
140	Ego07	13FE3-143	Maloca	F	E	1	Y
141	Withdrawn	10FD1-144	Maloca	F	D	1	Y
142	Withdrawn	12MD3-145	Maloca	M	D	1	Y
143	Ego69	18FH3-146	Maloca	F	H	1	Y
144	Withdrawn	8FC3-147	Maloca	F	C	1	Y
145	Ego61	13FE1-148	Maloca	F	E	1	Y
146	Ego02	12ME3-149	Maloca	M	E	1	N
147	Ego66	10FG3-150	Maloca	F	G	1	Y
148	Withdrawn	5FC2-151	Maloca	F	C	3	Y
149	Ego51	6FD3-153	Maloca	F	D	1	Y
150	Withdrawn	12FC3-155	Maloca	F	C	1	Y
151	Withdrawn	12FC3-156	Maloca	F	C	1	Y
152	Withdrawn	10FE3-158	Maloca	F	E	1	Y
153	Ego56	1FG3-160	Maloca	F	G	1	Y
154	Ego28	24MF3-161	Maloca	M	F	1	Y
155	Ego74	8FD3-162	Maloca	F	D	1	Y
156	Ego42	10FF1-163	Maloca	F	F	1	Y
157	Ego21	26FF1-164	Maloca	F	F	1	Y
158	Ego102	6FC3-165	Maloca	F	C	1	Y
159	Ego105	25FD1-166	Maloca	F	D	1	Y
160	Ego58	8FD3-168	Maloca	F	D	1	Y
161	Ego76	12FF3-169	Maloca	F	F	1	Y
162	Ego97	6FG5-170	Maloca	F	G	1	Y
163	Ego63	8FFX-171	Maloca	F	F	1	Y
164	Withdrawn	8FF1-172	Maloca	F	F	1	Y
165	Ego01	14FH5-173	Maloca	F	H	1	Y
166	Ego18	14FE1-174	Maloca	F	E	1	Y
167	Ego60	8MG3-175	Maloca	M	G	1	Y
168	Ego47	8ME3-176	Maloca	M	E	1	Y
169	Ego12	10FH5-177	Maloca	F	H	1	Y
170	Ego91	12FC3-178	Maloca	F	C	1	Y
171	Ego16	21FH1-179	Maloca	F	H	5	Y
172	Ego23	14FD3-180	Maloca	F	D	5	Y
173	Ego81	3FF3-181	Maloca	F	F	1	Y
174	Ego29	3FF3-182	Maloca	F	F	1	Y

175	Ego22	8MG3-184	Maloca	M	G	1	Y
176	Withdrawn	8FF3-185	Maloca	F	F	1	Y
177	Ego67	8FG5-186	Maloca	F	G	1	Y
178	Ego31	8FC1-187	Maloca	F	C	1	Y
179	Ego24	8FC3-188	Maloca	F	C	1	Y
180	Ego95	8FE1-189	Maloca	F	E	1	Y
181	Ego06	25FD3-190	Maloca	F	D	1	Y
182	Ego40	10FE1-191	Maloca	F	E	1	Y
183	Ego79	8FE3-193	Maloca	F	E	1	Y
184	Ego30	6FH3-194	Maloca	F	H	1	Y
185	Ego62	10FH3-195	Maloca	F	H	2	Y
186	Ego55	6FD3-196	Maloca	F	D	7	Y
187	Ego98	8FD3-199	Maloca	F	D	1	Y
188	Ego88	8FD1-200	Maloca	F	D	1	Y
189	Ego83	8FE3-201	Maloca	F	E	1	Y
190	Withdrawn	10FD2-202	Maloca	F	D	1	Y
191	Withdrawn	13FD3-203	Maloca	F	D	1	Y
192	Withdrawn	12MC3-204	Maloca	M	C	1	Y
193	Ego92	12FI1-205	Maloca	F	I	1	Y
194	Ego15	17FDX-206	Maloca	F	D	1	Y
195	Withdrawn	12FD3-207	Maloca	F	D	1	Y
196	Withdrawn	8FF1-208	Maloca	F	F	1	Y
197	Withdrawn	8FD2-209	Maloca	F	D	1	Y
