# Supplementary material for: Food sharing networks in a multi-ethnic, predominantly indigenous peri-urban community in the Brazilian Amazon
Source: PLoS One. 2026 Nov 11;21(11):e0360629. doi: 10.1371/journal.pone.0360629 (PMC13754111; doi:10.1371/journal.pone.0360629)
Supplement: Supplementary file 3 [file pone.0360629.s003.pdf]

Escala Brasileira de Segurança Alimentar – Version/Versão 2020 – SIMPLIFIED VERSION /VERSÃO SIMPLIFICADA

Please answer each question with "Yes" or "No"

Por gentileza para cada pergunta responda com "Sim ou "Não"

PERGUNTA	YES/ SIM	NO / NÃO
**FS1**- Were you or your relatives worried that the food would run out before you could get more?<br>Você e seus parentes ficaram preocupados de que acabasse a comida antes de conseguir mais?<br>Indé ne anamaitá tapitá saciára ubá resewára rewau timbiú?		
**FS2-** Did the food run out before you could get more?<br>Acabou a comida antes de você conseguir mais?<br>Amuramé ubá timbiú tipukusáre rewasému?		
**FS3-** Did you and your relatives NOT have conditions to have good and varied food?<br>Você e seus parentes NÃO tiveram condições de ter comida boa e variada?<br>Penhé ĩti periku pembaú timbiú purangawa amurupi?		
**FS4-** Did you and your relatives eat leftover food because you didn't have the means to get something else?<br>Você e seus parentes comeram as comidas que sobraram porque não tinham como conseguir outras coisas?<br>Penhé pembaú timbiú remiréra porque tímayé pewasému amunugá?		
**FS5**- Did you or another adult relative miss ONE meal because you didn't have the means to get it?<br>Você ou outro parente adulto ficou sem UMA refeição porque não tinha como conseguir?<br>Indé amú yanamaitá tuyuitá né yepé timbiú porque timayé pewasému?		
**FS6**- Did you or another adult relative eat less food than you wanted because you didn't have the means to get it?<br>Você ou outro parente adulto comeu menos comida do que queria porque não tinha como conseguir?<br>Indé amú yanamaitá tuyuitá tambaú mirí porque timayé tawasému?		
**FS7**- Did you and your adult relatives feel hungry but didn't eat because you didn't have the means to get food?<br>Você e seus parentes adultos sentiram fome, mas não comeram, porque não tinham como conseguir comida?<br>Indé amú yanamaitá tuyuitá pesã yumasí, títa ambaú, porque timayé pewasému timbiú?		
**FS8**- Did you or your adult relatives go a Whole day without eating because you didn't have the means to get food?<br>Você ou seus parentes adultos ficaram um dia inteiro sem comer porque não tinham como conseguir comida?<br>Indé amú yanamaitá pesasá yepé ára tipembaú porque timayé pewasému timbiú?		
**FS9*-** Did the children not have good and varied food because you didn't have the means to get food?<br>As crianças não tiveram comida boa e variada porque não tinha como conseguir comida?<br>Tainaitá titarikú timbiú puranga yumunáywa porque timayé pewasému?		
**FS10*-** Did any child eat less than they should have because you didn't have the means to get enough food?<br>Alguma criança comeu menos do que devia porque não tinha como conseguir o suficiente?<br>A mú tainaitá Tambaú mirí marã timayé pewasému amú turusú?		
**FS11*-** Did you give less food to a child because you didn't have the means to get more food?<br>Você deu menos comida para uma criança porque não tinha como conseguir mais comida?<br>Indé remé mirí timbiú yepé taína porque timayé rewasému turusú timbiú?		

**FS12*-** Did any child not have a meal during the day because you didn't have the means to get food?<br>Alguma criança ficou sem uma refeição durante o dia porque não tinha como conseguir comida?<br>Amú taína upitá né yepé timbiú yepé ára porque timayé rewasému timbiú?		
**FS13*-** Did any child feel hungry but didn't eat because you didn't have the means to get food?<br>Alguma criança ficou com fome mas não comeu porque não tinha como conseguir comida?<br>Amú taína upitá yumasí marã porque timayé rewasému timbiú?		
**FS14*-** Did any child go a whole day without eating, or only ate one time during the day, because you didn't have the means to get food?<br>Alguma criança ficou um dia inteiro sem comer, ou comeu somente uma vez durante o dia, porque não tinha como conseguir comida?<br>Amú tainaitá upitá yepé ára até karúka tiumbaú, ó umbá yepé viáji kuemaitá, porque timayé rewasému?		

* - Items FS9-14 should be Applied to households with children under 18 years old /Itens FS9-FS14 deveriam ser aplicados apenas aos domicílios com um menor de idade (< 18 anos).

Classification: Famillies with children <18 yrs
Classificação: Famílias com menores de 18 anos

- Food Secure / Segurança alimentar (0 points/ pontos)
- Light Food Insecurity / Insegurança alimentar leve (1 to/a 5 points / pontos)
- Moderate Food Insecurity / Insegurança alimentar moderada (6 to/a 9 points / pontos)
- Severe Food Insecurity / Insegurança alimentar grave (10 to/a 14 points / pontos)

Classification: Families without children <18 yrs
Classificação: Famílias sem menores de 18 anos

- Food Security /Segurança alimentar (0 points/pontos)
- Light Food Insecurity/Insegurança alimentar leve (1 to/a 3 points/pontos)
- Moderate Food Insecurity/Insegurança alimentar moderada (4 to/a 6 points/pontos)
- Severe Food Insecurity/Insegurança alimentar grave (7 to/a 8 points/pontos)
