# Supplementary material for: Food sharing networks in a multi-ethnic, predominantly indigenous peri-urban community in the Brazilian Amazon
Source: PLoS One. 2026 Nov 11;21(11):e0360629. doi: 10.1371/journal.pone.0360629 (PMC13754111; doi:10.1371/journal.pone.0360629)
Supplement: Supplementary file 4 [file pone.0360629.s004.pdf]

APPENDIX E: EGOCENTRIC NETWORK DATA
Parque das Tribos Egocentric Network Analysis
INTEGRATED Questionnaire
Harold Wright
(The Ohio State University)

Version: March 2, 2022

Parte ID. Participant ID / Identidade do Participante

ID1. Date/time	________________	ID4. Assistant	________________
ID2. Respondent	________________	ID5. Home address (min. street)	________________
ID3. Interviewer	________________	ID6. Location of Interview	________________

Reimbursement paid: Yes / No Envelope # ___

Interview Disclaimer Complete: Yes ☐ No ☐

____

Interviewer Signature I Date

A8. Interview conducted primarily in: Portuguese

A7. Interviewer Notes:

____

Seção EN: Redes Egocêntricos

Uma das principais questões da nossa pesquisa tem a ver com as relações sociais dentro da comunidade. Todos os nomes citados, assim como outras informações obtidas neste levantamento, são confidenciais. Estamos solicitando essas informações apenas para que possamos entender os padrões gerais das relações sociais relacionadas à alimentação dentro da comunidade. Todas as informações serão codificadas para garantir a anonimidade. Jamais revelaremos informações específicas sobre você ou outras pessoas em relatórios, artigos ou apresentações.

Parte 1. "Nos itens seguintes, vou pedir para você fazer uma relação dos nomes das pessoas com quem você interage de maneiras diferentes."

1. Nos últimos seis (06) meses, quem deu comida ***PARA VOCÊ OU ALGUÉM EM SUA CASA?***

Quem respondeu: ______________________________

Nome do/da Alter:
Intérpretes dos/das Alteres

NG1. ______________________________ NI1. ______________________________

NG2. ______________________________ NI2. ______________________________

NG3. ______________________________ NI3. ______________________________

NG4. ______________________________ NI4. ______________________________

NG5. ______________________________ NI5. ______________________________

NG6. ______________________________ NI6. ______________________________

NG7. ______________________________ NI7. ______________________________

NG8. ______________________________ NI8. ______________________________

NG9. ______________________________ NI9. ______________________________

NG10. ______________________________ NI10 . ______________________________

1= um vez em 06 meses; 2= duas vezes em 06 meses; 3= três vezes ou mais em 06 mesesn once a week; 6=once a week or more)

2. Nos últimos seis meses, ***PARA QUEM*** vocês deram comida?

Quem respondeu: ______________________________

Nome do/da Alter:

NG1. ______________________________

NG2. ______________________________

NG3. ______________________________

NG4. ______________________________

NG5. ______________________________

NG6. ______________________________

NG7. ______________________________

NG8. ______________________________

NG9. ______________________________

NG10. ______________________________

Intérpretes dos/das Alteres

NI1. ______________________________

NI2. ______________________________

NI3. ______________________________

NI4. ______________________________

NI5. ______________________________

NI6. ______________________________

NI7. ______________________________

NI8. ______________________________

NI9. ______________________________

NI10 . ______________________________

1= um vez em 06 meses; 2= duas vezes em 06 meses; 3= três vezes ou mais em 06 mesesn once a week; 6=once a week or more)

3. Nos últimos seis (06) meses, quem deu dinheiro ***PARA VOCÊ OU ALGUÉM EM SUA CASA?***

Quem respondeu: ______________________________

**Nome do/da Alter:**	**Intérpretes dos/das Alteres**
NG1. ______________________________	NI1. ____
NG2. ______________________________	NI2. ____
NG3. ______________________________	NI3. ____
NG4. ______________________________	NI4. ____
NG5. ______________________________	NI5. ____
NG6. ______________________________	NI6. ____
NG7. ______________________________	NI7. ____
NG8. ______________________________	NI8. ____
NG9. ______________________________	NI9. ____
NG10. ______________________________	NI10 . ____

1= um vez em 06 meses; 2= duas vezes em 06 meses; 3= três vezes ou mais em 06 mesesn once a week; 6=once a week or more)

______________	______	______________	______	______________	______	______________	______
______________	______	______________	______	______________	______	______________	______
______________	______	______________	______	______________	______	______________	______
______________	______	______________	______	______________	______	______________	______
______________	______	______________	______	______________	______	______________	______
______________	______	______________	______	______________	______	______________	______
______________	______	______________	______	______________	______	______________	______

4. Nos últimos seis meses, ***PARA QUEM*** vocês deram dinheiro?

Quem respondeu: ______________________________

**Nome do/da Alter:**		**Intérpretes dos/das Alteres**	
NG1.	______________________________	NI1.	______________________________
NG2.	______________________________	NI2.	______________________________
NG3.	______________________________	NI3.	______________________________
NG4.	______________________________	NI4.	______________________________
NG5.	______________________________	NI5.	______________________________
NG6.	______________________________	NI6.	______________________________
NG7.	______________________________	NI7.	______________________________
NG8.	______________________________	NI8.	______________________________
NG9.	______________________________	NI9.	______________________________
NG10.	______________________________	NI10 .	______________________________

1= um vez em 06 meses; 2= duas vezes em 06 meses; 3= três vezes ou mais em 06 mesesn once a week; 6=once a week or more)

5. Nos últimos seis (06) meses, quem emprestou equipamentos de caça ou pesca ***PARA VOCÊ OU ALGUÉM EM SUA CASA?***

Quem respondeu: ______________________________

**Nome do/da Alter:**		**Intérpretes dos/das Alteres**	
NG1.	______________________________	NI1.	______________________________
NG2.	______________________________	NI2.	______________________________
NG3.	______________________________	NI3.	______________________________
NG4.	______________________________	NI4.	______________________________
NG5.	______________________________	NI5.	______________________________
NG6.	______________________________	NI6.	______________________________
NG7.	______________________________	NI7.	______________________________
NG8.	______________________________	NI8.	______________________________
NG9.	______________________________	NI9.	______________________________
NG10.	______________________________	NI10 .	______________________________

1= um vez em 06 meses; 2= duas vezes em 06 meses; 3= três vezes ou mais em 06 mesesn once a week; 6=once a week or more)

6. Nos últimos seis meses, ***PARA QUEM*** vocês emprestaram equipamentos de caça ou pesca?

Quem respondeu: ______________________________

**Nome do/da Alter:**		**Intérpretes dos/das Alteres**	
NG1.	______________________________	NI1.	______________________________
NG2.	______________________________	NI2.	______________________________
NG3.	______________________________	NI3.	______________________________
NG4.	______________________________	NI4.	______________________________
NG5.	______________________________	NI5.	______________________________
NG6.	______________________________	NI6.	______________________________
NG7.	______________________________	NI7.	______________________________
NG8.	______________________________	NI8.	______________________________
NG9.	______________________________	NI9.	______________________________
NG10.	______________________________	NI10 .	______________________________

1= um vez em 06 meses; 2= duas vezes em 06 meses; 3= três vezes ou mais em 06 mesesn once a week; 6=once a week or more)

7. Nos últimos seis (06) meses, quem ajudou ***VOCÊ OU ALGUÉM EM SUA CASA*** com o plantio (incluindo emprestando ferramentas/dando plantas)?

Quem respondeu: ______________________________

**Nome do/da Alter:**		**Intérpretes dos/das Alteres**	
NG1.	______________________________	NI1.	______________________________
NG2.	______________________________	NI2.	______________________________
NG3.	______________________________	NI3.	______________________________
NG4.	______________________________	NI4.	______________________________
NG5.	______________________________	NI5.	______________________________
NG6.	______________________________	NI6.	______________________________
NG7.	______________________________	NI7.	______________________________
NG8.	______________________________	NI8.	______________________________
NG9.	______________________________	NI9.	______________________________
NG10.	______________________________	NI10 .	______________________________

1= um vez em 06 meses; 2= duas vezes em 06 meses; 3= três vezes ou mais em 06 mesesn once a week; 6=once a week or more)

8. Nos últimos seis meses, ***PARA QUEM*** vocês deram ajuda com o plantio (incluindo emprestando ferramentas/dando plantas)?

Quem respondeu: ______________________________

**Nome do/da Alter:**	**Intérpretes dos/das Alteres**
NG1. ______________________________	NI5. ______________________________
NG2. ______________________________	NI6. ______________________________
NG3. ______________________________	NI7. ______________________________
NG4. ______________________________	NI8. ______________________________
NG5. ______________________________	NI5. ______________________________
NG6. ______________________________	NI6. ______________________________
NG7. ______________________________	NI7. ______________________________
NG8. ______________________________	NI8. ______________________________
NG9. ______________________________	NI9. ______________________________
NG10. ______________________________	NI10 . ______________________________

1= um vez em 06 meses; 2= duas vezes em 06 meses; 3= três vezes ou mais em 06 mesesn once a week; 6=once a week or more)
