# Supplementary material for: Food sharing networks in a multi-ethnic, predominantly indigenous peri-urban community in the Brazilian Amazon
Source: PLoS One. 2026 Nov 11;21(11):e0360629. doi: 10.1371/journal.pone.0360629 (PMC13754111; doi:10.1371/journal.pone.0360629)

S5 Figure. Graphs of Socioeconomic Variables and Perception of Food Security (PDF)

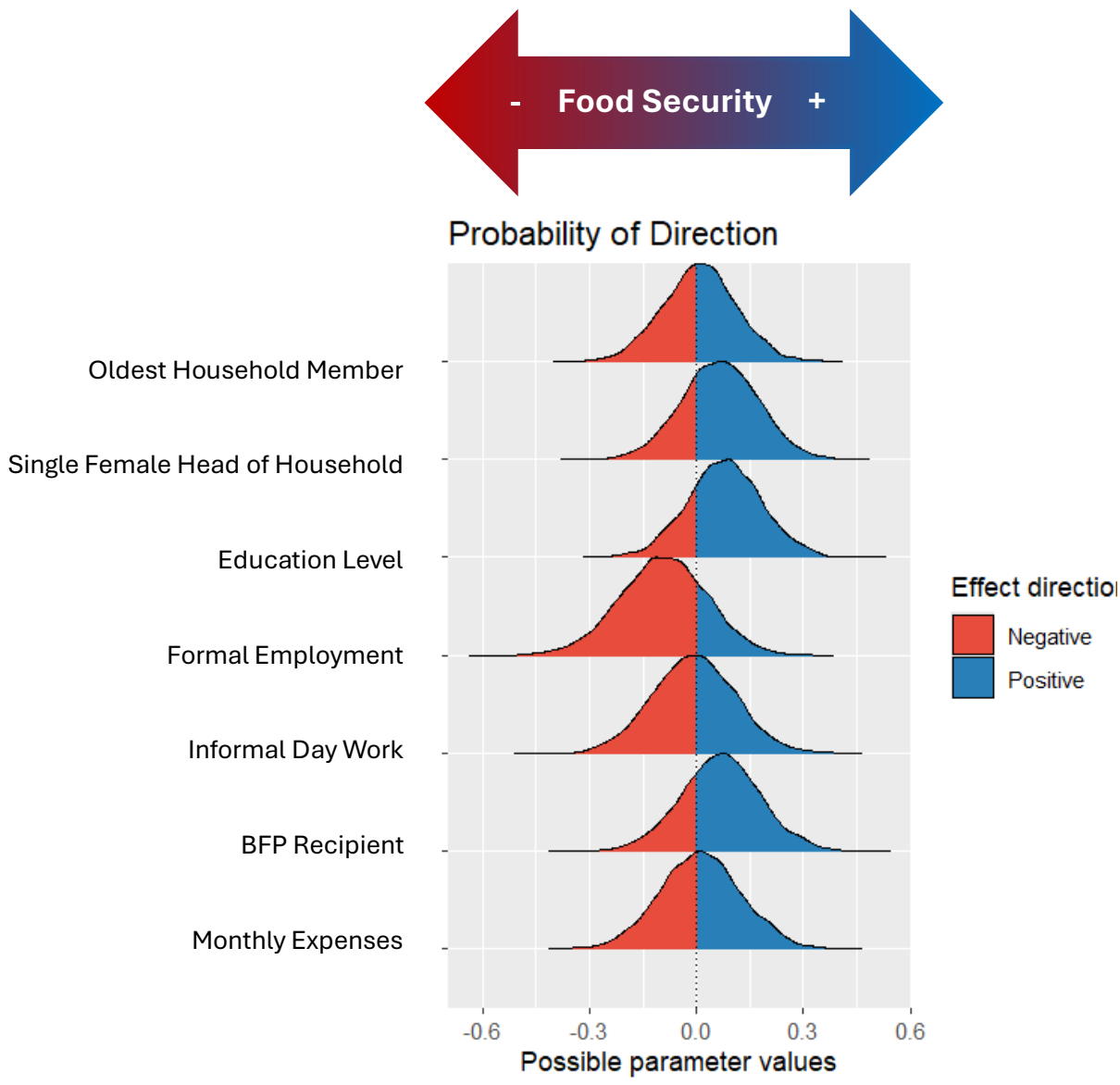

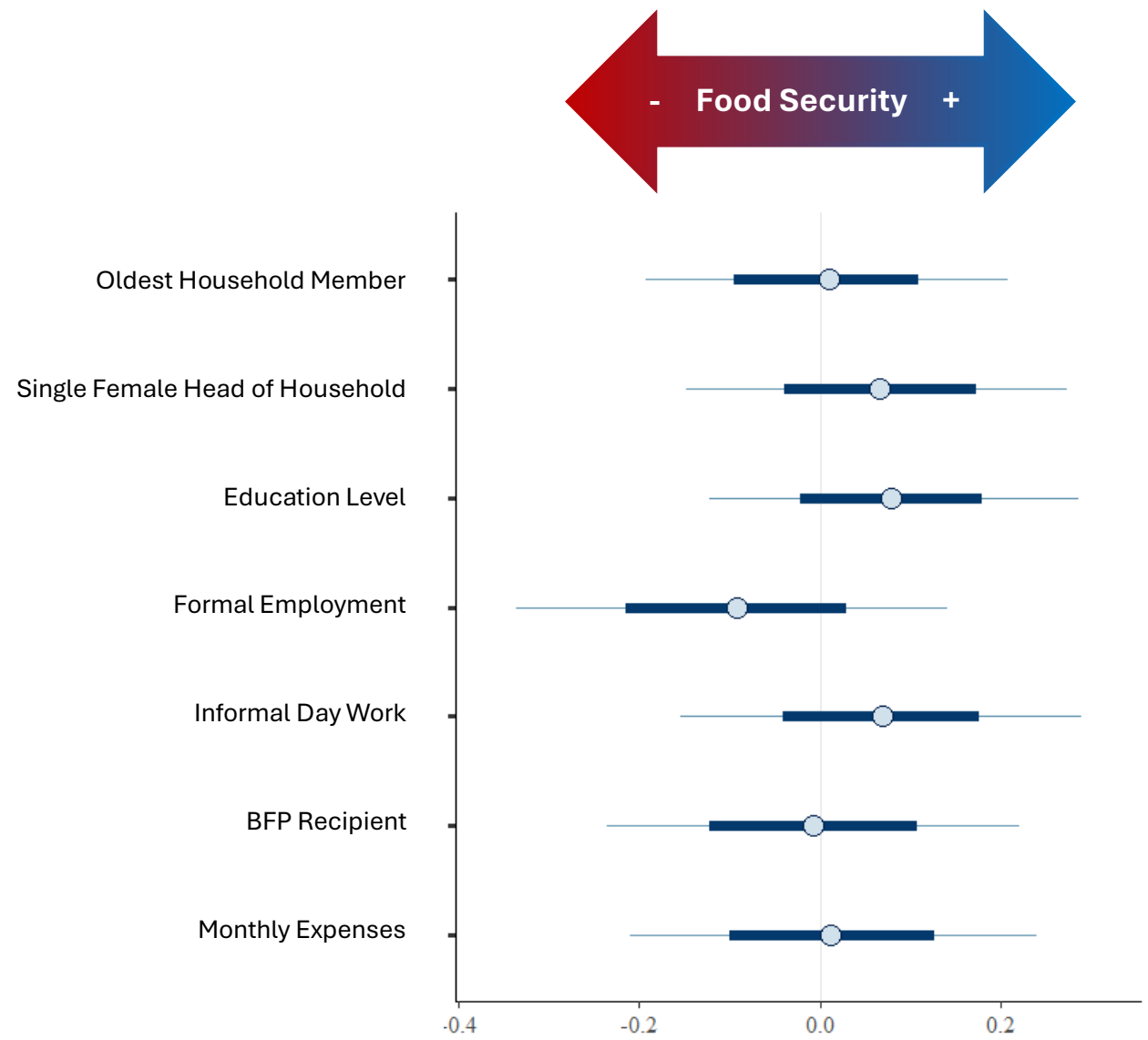

Supplement: Supplementary file 5 [file pone.0360629.s007.pdf]
