# Supplementary material for: Food sharing networks in a multi-ethnic, predominantly indigenous peri-urban community in the Brazilian Amazon
Source: PLoS One. 2026 Nov 11;21(11):e0360629. doi: 10.1371/journal.pone.0360629 (PMC13754111; doi:10.1371/journal.pone.0360629)
Supplement: Supplementary file 7 [file pone.0360629.s012.pdf]

Dear *Ecology of Food and Nutrition,*

We want to thank the two reviewers for their time and thoughtful consideration of our manuscript entitled "Food sharing networks in a multi-ethnic, predominantly indigenous peri-urban community in the Brazilian Amazon."

To each of the editor's and reviewers' comments we provide a brief response and then direct reviewers to the section and page(s) of the manuscript where we address the concern.

Editor comments (alterations to the manuscript are highlighted in yellow):

1. *Please ensure that your manuscript meets PLOS ONE's style requirements, including those for file naming.*

   Response: We have ensured that the document adheres to the PLOS ONE style requirements, as indicated in the corresponding style templates.

2. *Please include a complete copy of PLOS' questionnaire on inclusivity in global research in your revised manuscript. Our policy for research in this area aims to improve transparency in the reporting of research performed outside of researchers' own country or community. The policy applies to researchers who have travelled to a different country to conduct research, research with Indigenous populations or their lands, and research on cultural artefacts. The questionnaire can also be requested at the journal's discretion for any other submissions, even if these conditions are not met..*

   Response: We have completed the required questionnaire and have included it as a supplementary file with the manuscript, a PDF copy of which is additionally included as an annex to this letter.

3. *We note that the grant information you provided in the 'Funding Information' and 'Financial Disclosure' sections do not match. When you resubmit, please ensure that you provide the correct grant numbers for the awards you received for your study in the 'Funding Information' section..*

   Response: We have cross-checked the grant numbers and made the corrections, as indicated in the "Funding Information" section.

4. *Please amend your authorship list in your manuscript file to include author Harold Martin Wright III, Barbara A. Piperata, Sean S. Downey, Shane A. Scaggs.*

Response: We have amended the authorship list in the manuscript file as indicated, as well as including the name of *Dr. Therezinha de Jesus Pinto Fraxe*, as requested by a reviewer.

5. *Please amend the manuscript submission data (via Edit Submission) to include author HM Wright III, BA Piperata, SS Downey, and SA Scaggs.*

   Response: We have amended the manuscript submission data as indicated, as well as including the name of *TP Fraxe*, as requested by a reviewer.

6. *If the reviewer comments include a recommendation to cite specific previously published works, please review and evaluate these publications to determine whether they are relevant and should be cited. There is no requirement to cite these works unless the editor has indicated otherwise.*

   Response: We have amended the authorship list in the manuscript file as indicated, as well as including the name of *TP Fraxe*, as requested by a reviewer.

Reviewer 1 (alterations to the manuscript are in blue)

(No alterations were indicated by the reviewer).

Reviewer 2 (alterations to the manuscript are in blue):

1. *The name of the "principal investigator" and/or the "public contact" registered with CONEP does not match any of the manuscript's authors.*

   Response: The principal investigator and public contact, *Dr. Therezinha de Jesus Pinto Fraxe* of the Federal University of Amazonas (UFAM) is associated with the macro project entitled "C&T AGROFLORESTA: Uso de tecnologias para a redução do desmatamento e inclusão socioprodutiva de comunidades rurais em situação de vulnerabilidade socioeconômica," under which this project is included, has reviewed the article in question and has accepted inclusion as co-author.

2. *The data collection dates described in the manuscript occur after the period authorized in the protocol registered on Plataforma Brasil.*

   Response: The project was originally included in the CAAE and protocol indicated in the draft of the article with the original intent to complete fieldwork in 2021. However, due to the COVID-19 pandemic, it was transferred to the project indicated in the updated draft with a copy of the authorization and letter of clarification included as a supplementary file; however, the original CAAE and protocol were inadvertently included in the original draft due to an oversight by the lead author. It has since been

updated, with the accurate chronology of interviews related to this article as substantiated by the annexed letters and highlighted in the text.

3. *To ensure ethical compliance and proper alignment between the study presented and the approved project, I kindly request the following clarifications:*
   How the study described in the manuscript relates to the approved protocol whose number was provided.
   Response: The study relates to the current macro project indicated in the current manuscript in that it investigates land resource use and social inclusion as a means of sustainable land resource use and social inclusion by Indigenous and riparian migrant groups living on the urban periphery of that Amazonian city.
   What role the authors played in the approved project, considering that their names do not appear as the principal investigator or public contact.
   Response: The authors fulfilled the following roles:
   a. Conceptualization: Harold Wright, Sean Downey, Barbara Piperata
   b. Data curation: Harold Wright
   c. Formal analysis: Harold Wright , Shane Scaggs
   d. Funding acquisition: Harold Wright
   e. Investigation: Harold Wright
   f. Methodology: Harold Wright, Barbara Piperata, Sean Downey
   g. Visualization: Harold Wright, Shane Scagg
   h. Supervision: Barbara Piperata (USA), Therezinha de Jesus Pinto Fraxe (Brazil)
   i. Writing – original draft: Harold Wright
   j. Writing – review and editing: Harold Wright, Barbara Piperata, Sean Downey, Shane Scaggs, Therezinha de Jesus Pinto Fraxe

   How the discrepancy between the data collection period described in the manuscript and the period authorized by the CEP/CONEP is explained.

   Response: The project was originally included in the CAAE and protocol indicated in the draft of the article with the original intent to complete fieldwork in 2021. However, due to the COVID-19 pandemic, it was transferred to the project indicated in the updated draft; however, the original CAAE and protocol were inadvertently included in the original draft due to an oversight by the lead author. The ethical approval has since been updated, as substantiated by the annexed letters.

   Additionally, I request the submission of supplementary documentation, such as: a clarification letter signed by the principal investigator listed in the protocol; proof that the authors were part of the approved research team; an amendment approved by the CEP/CONEP including new team members and extending the data collection period.

   Response: The requested supplementary documentation is included in the annexes attached to this letter.

We remain at your disposal for any further clarifications or questions.

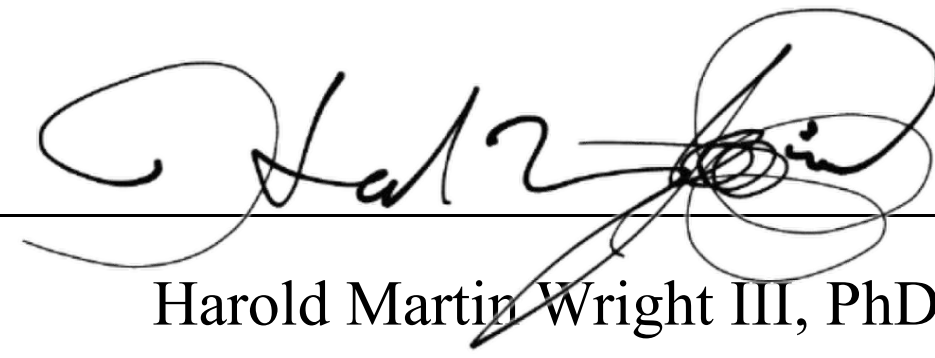

Harold Martin Wright III, PhD
Corresponding Author

Inclusivity in global research

PLOS' policy on inclusivity in global research aims to improve transparency in the reporting of research performed outside of researchers' own country or community and ensures that PLOS publications reporting global research adhere to high standards for research ethics and authorship. Authors of relevant research articles may be asked to complete the questionnaire below, which outlines ethical, cultural, and scientific considerations specific to inclusivity in global research. This questionnaire may be requested when researchers have travelled to a different country to conduct research, if research uses samples collected in another country, research with Indigenous populations or their lands, or if research is on cultural artefacts. Researchers travelling to another country solely to use laboratory equipment will not normally be required to complete the questionnaire. However, the questionnaire can be requested at the journal's discretion for any submission – if you have been requested to complete this questionnaire by the PLOS journal you submitted to, please do so.

Please complete the questionnaire below and include this as a Supporting Information file with your manuscript. Note that if your paper is accepted for publication, this checklist will be published with your article in the supporting information files. Please ensure that you reference the checklist in the main body of your manuscript. We suggest adding a subsection 'Inclusivity in global research' to your Methods section and adding the following sentence: "Additional information regarding the ethical, cultural, and scientific considerations specific to inclusivity in global research is included in the Supporting Information (SX Checklist)"

The questions have been designed to be applicable to a wide range of study types, and there are subsections for both human subjects research and non-human subjects research. If any of the questions are not relevant to your research please mark them as "N/A" as appropriate.

Ethical considerations, permits and authorship

This section is applicable to all research types.

Provide details as to who granted permissions and/or consent for the study to take place in the Methods section of your manuscript. This should include the names of **all** ethics boards, governmental organizations, community leaders or other bodies that provided approval for the study. If individuals provided approval refer to these people by their role or title but do not list their name(s).

Reported on page number: 9

If there were any deviations from the study protocol after approval was obtained please provide details of these changes in the Methods section of your manuscript.

Reported on page number: 9

Did this study involve local collaborators that are residents of the country where the research was conducted or members of the community studied? If you do not have any authors from said communities, please provide an explanation for this below.

This study involved two Brazilian researchers, Dr. Harold Martin Wright III and Dr. Therezinha de Jesus Pinto Fraxe, both affiliated with the Universidade Federal do Amazonas (UFAM).

Everyone listed as an author should meet PLOS' criteria for authorship and all individuals who meet these criteria should be included in the author byline, rather than the acknowledgements. For further information please see the journal's Authorship Policy.

Human subjects research (e.g. health research, medical research, cross-cultural psychology)

Did you obtain written informed consent from a representative of the local community or region before the research took place? How did you establish who speaks for the community? Details of written informed consent obtained from study participants should be reported separately in the Methods section of your manuscript.

Written informed consent was obtained from all study participants.

How did members of the local community provide input on the aims of the research investigation, its methodology, and its anticipated outcome(s)?

Focus group interviews were conducted previous to the study to gauge interest and obtain local imput.

When engaging with the local community, how did you ensure that the informed consent documents and other materials could be understood by local stakeholders?

All informed consent documents were available in both Portuguese, as well as in the Nheengatu languages for those requiring it, and all participants indicated that they understood completely the aims and function of the research activities.

Will the findings of the research be made available in an understandable format to stakeholders in the community where the study was conducted (e.g. via a presentation, summary report, copies of publications, etc.)? Please provide details of how this will be achieved.

Initial summary reports have been provided to local leaders, and a full presentation of the results will be provided to all interested community residents upon the lead author's next visit to Brazil.

Non-human subjects research using specimens/ animals collected as part of the study, or those housed in archival collections. Examples include archaeology, paleontology, botany and zoology.

Did the permission you obtained from a local authority to perform the study include an agreement on access to outputs and benefit sharing? This may include procedures to enable fair distribution of the benefits and resources arising from the research performed. Please include any details of Prior Informed Consent and Benefit Sharing Agreements obtained. These may be required by field-specific regulations, for example the Convention on Biological Diversity (CBD) and the associated Nagoya Protocol.

No non-human subject research or biological specimens were obtained as a result of this research. The informed prior consent form is included as S9 File.

If the material used in your study was imported, please A) provide the year it was imported and B) indicate whether permits were obtained to import/export the materials used, C) provide details of any permits obtained. If this information is not available, please indicate this.

No material was imported.

If you used archival specimens, please state how the material used in your study was acquired by the institute it is held in and provide details of any permits obtained for the original excavations/ sample collection. If this information is not available, please indicate this.

No archival specimens were used.

How was the potential cultural significance of the materials collected in your study to local communities considered in your research design? Were Indigenous peoples and/or local researchers and institutions involved with archaeological excavations / collection of specimens? If so, please provide a description of their involvement.

No archaeological excavations or collection of specimens were obtained as part of this research.

If your manuscript includes photographs of human remains please indicate whether authors obtained permission from descendants or affiliated cultural communities to do so.

No photographs of human subjects or human remains are included as part of or are relevant to this research.

A quem possa interessar:

Declaro para os devidos fins que o projeto de pesquisa intitulado **“Segurança Alimentar, Identidade e Redes Egocêntricas entre os moradores indígenas de Manaus, Amazonas, Brasil”**, realizado pelo pesquisador visitante Harold Martin Wright III, PhD. da Universidade Estadual de Ohio, cidadão brasileiro, casado, portador do CPF n. 534.610.612-04, RG n.4050454-9 SSP/AM, durante o período compreendido entre 1º de maio de 2022 e 31 de outubro de 2022 na cidade de Manaus, Amazonas, foi originalmente integrado ao projeto de pesquisa “Cooperativismo como estratégia de inclusão socioeconômica e melhoria da qualidade de vida de comunidades rurais do Amazonas”, sob a coordenação da Profa. Dra. Therezinha de Jesus Pinto Fraxe, abaixo assinada, **CAAE: 24335319.2.0000.5020,** nos termos do parecer nº**:** 3.687.909.

Posteriormente, foi reintegrado ao projeto de pesquisa “C&T Agrofloresta: Uso de tecnologias para a redução do desmatamento e inclusão socioprodutiva de comunidades rurais em situação de vulnerabilidade socioeconômica”, sob a mesma coordenação da Profa. Dra. Therezinha de Jesus Pinto Fraxe, abaixo assinada, **autorizado pela Comissão Nacional de Ética em Pesquisa (CONEP)** para a realização de pesquisas com seres humanos (*veja página anexada*) com as relevantes atividades de pesquisa realizadas no período de 13/09/2022-30/10/2022. A reintegração do projeto foi necessária devido à pandemia da COVID-19, que atrasou o início das atividades para 2022.

Por ser expressão da verdade, firmo a presente declaração para os fins que se fizerem necessários.

Manaus, 23 de maio de 2026

Therezinha de Jesus Pinto Fraxe

Therezinha de Jesus Pinto Fraxe
Coordenadora do Projeto

To whom it may concern:

I hereby declare, for all due purposes, that the research project entitled *“Food Security, Identity, and Egocentric Networks among Indigenous Residents of Manaus, Amazonas, Brazil,”* conducted by the visiting researcher **HAROLD MARTIN WRIGHT III, PhD**, from The Ohio State University, a Brazilian citizen, married, holder of CPF No. 534.610.612-04 and RG No. 4050454-9 SSP/AM, during the period from May 1, 2022 to October 31, 2022 in the city of Manaus, Amazonas, was originally integrated into the research project *“Cooperativism as a Strategy for Socioeconomic Inclusion and Improvement of Quality of Life in Rural Communities of Amazonas,”* under the coordination of Prof. Dr. Therezinha de Jesus Pinto Fraxe, the undersigned, CAAE: 24335319.2.0000.5020, pursuant to Ethics Committee Opinion (Parecer) No. 3.687.909.

Subsequently, it was reintegrated into the research project *“C&T AGROFLORESTA: Uso de tecnologias para a redução do desmatamento e inclusão socioprodutiva de comunidades rurais em situação de vulnerabilidade socioeconômica”* (“S&T: The use of technologies for the reduction of deforestation and socio-productive inclusion of rural communities in situations of socio-economic vulnerability”), under the same coordination of Prof. Dr. Therezinha de Jesus Pinto Fraxe, the undersigned, **authorized by the National Commission for Ethics in Research** for research involving human subjects (*see attached*), with the relevant research activities undertaken in the period from 09/13/2022-10/30/2022. The reintegration of the project was necessary due to the COVID-19 pandemic, which delayed the start of field activities until 2022.

Being a true and accurate statement, I issue this declaration for all purposes for which it may be required.

Manaus, May 23, 2026

Therezinha de Jesus Pinto Fraxe

Project Coordinator

A quem possa interessar:

Venho, mui respeitosamente, por meio da presente, responder às indagações formuladas pelo(a) revisor(a) da revista acadêmica *PLOS One* referentes à submissão PONE-D-25-54642, intitulada "**Food sharing networks in a multi-ethnic, predominantly indigenous peri-urban community in the Brazilian Amazon**".

No que tange aos questionamentos apresentados, cumpre esclarecer que:

- O referido projeto integra o macroprojeto intitulado, "C&T AGROFLORESTA: Uso de tecnologias para a redução do desmatamento e inclusão socioprodutiva de comunidades rurais em situação de vulnerabilidade socioeconômica", sob a mesma coordenação da Profa. Dra. Therezinha de Jesus Pinto Fraxe, ora signatária, que foi autorizado para a realização da mencionada pesquisa com seres humanos, no âmbito da investigação de atividades cooperativas relacionadas à subsistência e à qualidade de vida em comunidade economicamente vulnerável situada fora do perímetro urbano, caracterizando-se, portanto, como comunidade rural (*veja página anexada*).
- O pesquisador visitante, Prof. Dr. Harold Martin Wright III, PhD., da Universidade Estadual de Ohio, cidadão brasileiro, casado, portador do CPF n. 534.610.612-04, RG n.4050454-9 SSP/AM, foi formalmente incluído na equipe de pesquisa do referido macroprojeto como meu orientado, conforme a carta que segue anexa.
- A participação dos demais pesquisadores Profa. Dra. Barbara Piperata, Prof. Dr. Sean Downey e Prof. Dr. Shane Scaggs, todos da Universidade Estatal de Ohio, restringe-se exclusivamente à revisão e à edição do texto e dos cálculos estatísticos. Vale destacar que os três coautores não tiveram, em nenhum momento, acesso a dados contendo informações pessoais e confidenciais dos participantes da pesquisa, tampouco estiveram presentes no trabalho de campo realizando coleta de dados.

- Na qualidade de pesquisadora principal e contato público do macroprojeto supracitado, declaro ter lido o manuscrito submetido à revista *PLOS One* e concordo com a inclusão de meu nome como coautora, ressaltando que o autor principal deve ser o pesquisador visitante, Prof. Dr. Harold Martin Wright III, PhD, responsável por toda a coleta de dados, análise e redação do corpo do artigo.

Sem outro assunto, subscrevo-me com elevada estima.

Manaus, 23 de maio de 2026

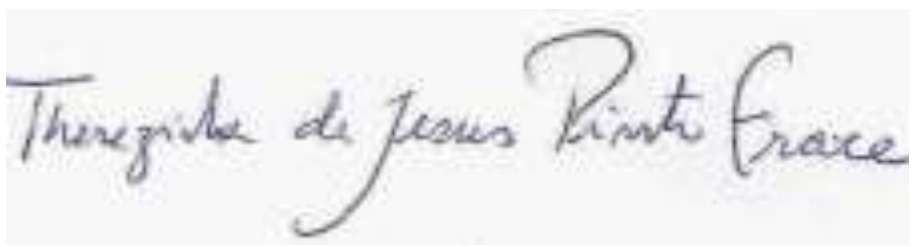

Therezinha de Jesus Pinto Fraxe
Coordenadora do Projeto

To whom it may concern:

I hereby, most respectfully, respond to the inquiries made by the reviewer of the academic journal PLOS One regarding submission PONED2554642, entitled *"Food sharing networks in a multi-ethnic, predominantly Indigenous peri-urban community in the Brazilian Amazon."*

With respect to the questions presented, I clarify the following:

The aforementioned project is part of the larger research project entitled *"C&T AGROFLORESTA: Uso de tecnologias para a redução do desmatamento e inclusão socioprodutiva de comunidades rurais em situação de vulnerabilidade socioeconômica"* ("S&T: The use of technologies for the reduction of deforestation and socio-productive inclusion of rural communities in situations of socio-economic vulnerability"), under the same coordination of Prof. Dr. Therezinha de Jesus Pinto Fraxe, the undersigned, authorized by the National Commission for Ethics in Research for research involving human subjects (*see attached*), which authorizes the conduct of the referenced research involving human subjects within the broader investigation of cooperative activities related to subsistence and quality of life in an economically vulnerable community located outside the urban perimeter, thus characterized as a rural community.

The visiting researcher, Prof. Dr. HAROLD MARTIN WRIGHT III, PhD, from The Ohio State University, a Brazilian citizen, married, CPF n. 534.610.612-04, RG n.4050454-9 SSP/AM, was formally included in the research team of the aforementioned macroproject as my advisee, as indicated in the attached letter.

The participation of the other researchers — Dr. Barbara Piperata, Dr. Sean Downey, and Dr. Shane Scaggs, all from The Ohio State University — is strictly limited to the revision and editing of the manuscript and statistical calculations. It is important to emphasize that these three coauthors did not, at any time, have access to data containing personal or confidential information about research participants, nor were they present during fieldwork or involved in data collection.

As the principal investigator and public contact for the above-mentioned macroproject, I hereby declare that I have read the manuscript submitted to PLOS One and agree with the inclusion of my name as a coauthor, noting that the lead author should be the visiting researcher, Prof. Dr. Harold Martin Wright III, PhD, who was responsible for all data collection, analysis, and writing of the manuscript.

With no further matters to address, I sign this letter with high regard.

Manaus, May 23, 2026

Therezinha de Jesus Pinto Fraxe

Project Coordinator

Manaus, 09 de janeiro de 2020

Estimado Comitê de Seleção da DDRA da Fulbright-Hays,

Venho, por meio desta carta, declarar meu apoio à proposta de pesquisa de Harold Martin Wright sobre *Redes sociais indígenas urbanas e seu papel na segurança alimentar*. Sou professor titular da Universidade Federal do Amazonas (UFAM) em Manaus, Brasil, e atuo como coordenadora do Núcleo de Socioeconomia (NUSEC/FCA/UFAM) e vice coordenadora do Programa de Pós-Graduação em Ciências do Ambiente e Sustentabilidade na Amazônia - PPGCASA. Venho acompanhando a trajetória de Harold como pesquisador há mais de doze anos, desde o início de sua carreira como pesquisador no Programa de Pós-Graduação em Ecologia no Instituto Nacional de Pesquisas da Amazônia ("Instituto Nacional de Pesquisas da Amazônia - INPA").

Compartilho os interesses de pesquisa de Harold nos campos da transculturalidade, sustentabilidade, agricultura familiar e redes sociais, além de estar disponível para aconselhá-lo e apoiá-lo durante seu período de pesquisas no Brasil. Esse apoio incluirá orientação sobre análise de redes sociais e sua aplicabilidade nos ecossistemas urbanos, assim como assistência na análise de dados transculturais e de sustentabilidade. Harold compreende que meu apoio será de natureza colaborativa, que ele é o único responsável pelo apoio logístico e financeiro durante seu trabalho de campo no Brasil e que a UFAM não fará contribuições desta natureza. O projeto de Harold é de crítica importância, dada a relativa falta de estudo sobre a segurança alimentar das populações indígenas urbanas na Amazônia brasileira. Além disso, nosso trabalho em conjunto contribuirá ainda mais para o crescimento vital da cooperação internacional entre os Estados Unidos e o Brasil, e quaisquer publicações subsequentes, como resultado dessa parceria refletirão essa colaboração.

Aguardo a chegada de Harold no Brasil para iniciar nosso trabalho conjunto e reitero meu entusiasmo e compromisso com este importante projeto. Garanto-lhe que Harold será bem recebido e procurarei fornecer as orientações necessárias para garantir o sucesso deste projeto em todos os níveis.

Atenciosamente,

Therezinha de Jesus Pinto Fraxe

Therezinha Jesus Pinto Fraxe

(English Translation of Affiliation Letter from Therezinha de Jesus Pinto Fraxe By: Harold Martin Wright III)

Dear Fulbright-Hays DDRA Selection Committee,

I am addressing this letter to you to declare my support for Harold Martin Wright's research proposal on *Urban Indigenous Social Networks and their Role in Food Security*. I am a tenured professor at the Federal University of Amazonas ("Universidade Federal do Amazonas – UFAM") in Manaus, Brazil, and am the coordinator of the Socioeconomic Center ("Núcleo de Socioeconomia – NUSEC/FCA/UFAM") and am vice-coordinator of the Graduate Program in Environmental and Sustainability Sciences in the Amazonas ("Programa de Pós-Graduação em Ciências do Ambiente e Sustentabilidade na Amazônia – PPGCASA"). I have followed Harold's trajectory as a researcher for over twelve years, since his early graduate work at the National Institute for Amazonian Research ("Instituto Nacional de Pesquisas da Amazônia – INPA").

I share Harold's research interests in the fields of transculturalism, sustainability, familial agriculture, and social networks, and am available to advise and support to him during his period of stay in Brazil. This support will include orientation on social network analysis and its applicability in urban ecosystems, as well as assistance with analysis of transcultural data and sustainability. Harold is aware that my support will be collaborative in nature, and that he alone is responsible for logistic and financial support during his fieldwork in Brazil, with UFAM not contributing to either of these categories. Harold's project is of critical importance, given the relative lack of study on food security among urban indigenous populations in the Brazilian Amazon. Furthermore, our work together will further contribute to the vital growth in international cooperation between the United States and Brazil, as any subsequent publications resulting from this collaboration will reflect this collaboration.

I look forward to Harold's arrival in Brazil to start our work together, and reiterate my enthusiasm and commitment to this project of importance. I assure you that Harold will be well-received, and that I will endeavor to provide the guidance necessary to ensure the success of this project at all levels.

Best Regards,
Therezinha de Jesus Pinto Fraxe

wright.2067@osu.edu •••••••• LOGIN

Esqueceu a senha? Cadastre-se v4.0.9_rc01

DETALHAR PROJETO DE PESQUISA

DADOS DO PROJETO DE PESQUISA

Título Público: C&T AGROFLORESTA: Uso de tecnologias para a redução do desmatamento e inclusão socioprodutiva de comunidades rurais em situação de vulnerabilidade socioeconômica
Pesquisador Responsável: Therezinha de Jesus Pinto Fraxe
Contato Público: Therezinha de Jesus Pinto Fraxe
Condições de saúde ou problemas estudados:
Descritores CID - Gerais:
Descritores CID - Específicos:
Descritores CID - da Intervenção:
Data de Aprovação Ética do CEP/CONEP: 31/08/2022

PLATAFORMA BRASIL COORDENADOR PLATAFORMA BRASIL

DADOS DA INSTITUIÇÃO PROPONENTE

Nome da Instituição: Universidade Federal do Amazonas - UFAM
Cidade: MANAUS

DADOS DO COMITÊ DE ÉTICA EM PESQUISA

Comitê de Ética Responsável: 5020 - Universidade Federal do Amazonas - UFAM
Endereço: Rua Teresina, 4950
Telefone: (92)3305-4000
E-mail: cep@ufam.edu.br

CENTRO(S) PARTICIPANTE(S) DO PROJETO DE PESQUISA

CENTRO(S) COPARTICIPANTE(S) DO PROJETO DE PESQUISA

Voltar

Chat
