# Supplementary material for: Food sharing networks in a multi-ethnic, predominantly indigenous peri-urban community in the Brazilian Amazon
Source: PLoS One. 2026 Nov 11;21(11):e0360629. doi: 10.1371/journal.pone.0360629 (PMC13754111; doi:10.1371/journal.pone.0360629)
Supplement: Supplementary file 8 [file pone.0360629.s013.pdf]

Dear *PLoS ONE* Editors and Reviewers,

We want to thank the two reviewers for their time and thoughtful consideration of our manuscript entitled “Food sharing networks in a multi-ethnic, predominantly indigenous peri-urban community in the Brazilian Amazon.”

To each of the editor’s and reviewers’ comments we provide a brief response and then direct reviewers to the section and page(s) of the manuscript where we address the concern. Specific Line Number locations are included under each of the reviewers’ respective comments.

Additionally, we made minor editorial revisions to improve clarity and streamline the presentation. No data or analytical results in the Methods, Results, or Discussion sections were altered.

Editor comments:

1. *Please address the following points:*

- ***Comment:*** *Ensure all claims regarding food sharing and food insecurity are framed strictly as cross-sectional associations and clarify the analytical treatment of the scaled EBIA variable.*

- **Response:** All statements linking food sharing, social network characteristics, and household food insecurity have been revised to ensure they are framed strictly as cross-sectional associations. Causal language has been removed throughout. We also clarified the analytical treatment of the EBIA variable in both the Methods and Discussion sections, including a statement noting that EBIA measures reported household experiences of food insecurity rather than dietary quality or nutritional status.

- ***Comment:*** *Verify that recruitment biases, response/refusal rates, local instrument adaptations, and VIF values are fully reported.*

- **Response:** Recruitment biases and gendered sampling patterns are now explicitly described. Response/refusal rate information is reported in S2 File and referenced in the text. Local EBIA adaptations are detailed in the Methods. VIF values are reported in the Results.

- ***Comment:*** *Avoid oversimplified binaries between social and economic capital, and ensure structural conditions remain central.*

- **Response:** We revised the Discussion to remove any binary framing between social and economic capital and now emphasize that the observed patterns represent context-specific associations rather than contrasts.

We also added a dedicated paragraph outlining the structural conditions shaping food access in Parque das Tribos, including irregular wage labor, limited formal employment, and reliance on conditional cash-transfer programs, to ensure that political-economic context remains central to the interpretation of results.

- ***Comment:*** *Confirm that de-identification procedures, participant consent for data release, and community-level authorizations are explicitly addressed to mitigate re-identification risks in small communities.*

- **Response:** We have fully addressed this point. The Methods section now explicitly describes our de-identification procedures, including the removal of names, household identifiers, and any potentially identifying relational information, as well as the use of randomized alphanumeric codes for all network nodes and visualizations.
  We also clarify that participants provided informed consent for data collection and use, and that approval for the study was obtained from Parque das Tribos community leadership.
  Finally, we added language noting that data were stored securely, shared only in aggregate form, and handled in accordance with principles of Indigenous data sovereignty to minimize re-identification risks in a small community.

- ***Comment:*** *Fix the inconsistencies in Figure 1, merge the isolated sentence at Line 124, and simplify Lines 127-129.*

- **Response:** All requested corrections have been completed. The inconsistencies in Figure 1 and its caption have been fixed to ensure alignment between the text, labels, and visual elements.
  The isolated sentence at Line 124 has been merged into the following paragraph to resolve the structural issue noted by the reviewer.
  Lines 127–129 have been simplified for clarity and improved readability.

Reviewer 3 (alterations to the manuscript are in blue and underlined, or purple and boldened/underlined in the case of alterations that address both Reviewers' comments)

We thank the Reviewer for these suggestions. We address each in turn as follows:

1. ***Comment:*** *Explicit Hypotheses: The introduction builds an excellent theoretical foundation, but the specific hypotheses being tested remain somewhat implicit. I suggest formally declaring the working hypotheses immediately following the three research questions, as this might provide a sharper and more direct analytical roadmap for the reader.*

   Response: In revising the *Introduction*, we replaced the original research questions with explicit, formally stated hypotheses, which now appear in the location the reviewer indicated

(see **Lines 151–185**). This revision provides a clearer analytical roadmap and makes the expectations guiding the study fully explicit. The bridge paragraph preceding the hypotheses was also refined to ensure a smooth conceptual transition.

2. ***Comment:*** *Ecological Context of Bridge Ties : The explanation that bridge ties facilitate indirect access to resources is statistically robust. However, I suggest adding one or two qualitative sentences in the discussion regarding what specific resources (e.g ., hunted, fished, and foraged items versus cash-purchased goods) typically flow through these interethnic networks. Please consider this possibility, as it could deeply enrich the biological and social interpretation of the network's "quality."*

   Response: Qualitative responses during the network interviews indicate that the resources circulating through these ties were ecologically diverse. Participants described exchanges involving prepared meals and staple ingredients but also items obtained through hunting, fishing, and small-scale foraging, as well as garden produce such as bananas, *macaxeira (Manihot esculenta Crantz)*, herbs used in teas, and *cheiro-verde (*a mix of parsley and scallions). These mixed flows of purchased and self-produced foods help explain why actors occupying bridge-tie positions reported higher network quality: they connect households to resource types that differ in origin, seasonality, and reliability (see **Lines 569-577**).

3. ***Comment:*** *In the Data Analysis section, the authors correctly mention checking for multicollinearity by calculating the Variance Inflation Factor (VIF). I suggest briefly stating the highest observed VIF value in the text, just to help explicitly confirm for the reader that multicollinearity remained strictly within acceptable limits for the modeling.*

   Response: We have added the highest observed VIF value directly to the Data Analysis section to make the assessment of multicollinearity fully explicit. As noted in the Results, all VIFs fell between 1.00 and 1.53, confirming that multicollinearity was well within acceptable limits for the regression models (see **Lines 310-312, 387–388**).

Reviewer 4 (alterations to the manuscript are in red and boldened, or purple and boldened/underlined in the case of alterations that address both Reviewers' comments):

1. ***Comment:*** *Distinguish food insecurity, nutrition, and "perception." EBIA measures reported household experiences of food insecurity; it does not measure dietary quality, nutrient adequacy, anthropometric status, or access to culturally preferred foods. The manuscript repeatedly moves from EBIA scores to claims that networks "improve," "mitigate," or "compensate for" food insecurity and concludes that social capital is more important than economic capital. These causal and comparative claims are not justified. I suggest to reframe these throughout the manuscript as cross-sectional associations with reported household food insecurity. Also, I'd suggest a further discussion on EBIA as a tool. Is EBIA only*

measuring "perception" ? Otherwise, the researchers may inadvertently discount participants' lived material experiences.

Response: Throughout the manuscript, we revised all statements linking social networks, food sharing, and household food insecurity to ensure they are framed strictly as cross-sectional associations with reported household food insecurity, as measured by the EBIA. All causal or comparative language (e.g., "improve," "mitigate," "compensate for," "more important than") has been removed (see **Lines 105–108**, **142–150**, **417-431**).

To address the reviewer's concern regarding EBIA's conceptual domain, we added explicit clarification in both the Methods and Discussion (see **Lines 259-271**, **444-471**). In the Discussion, we now state:

"*It is important to note that the EBIA captures reported household experiences of food insecurity rather than dietary quality, nutrient adequacy, or anthropometric status, and our interpretation reflects this distinction (20, 89, 115).*"

This sentence directly addresses the distinction between food insecurity, nutrition, and perception, and clarifies that EBIA measures experiential household food insecurity, not dietary intake, nutritional status, or access to culturally preferred foods.

We also expanded the Methods section to describe how EBIA was adapted for the local context, how categorical EBIA classifications were used to construct a comparable ordinal variable across household types, and how this variable was incorporated into regression models.

Together, these revisions ensure that the manuscript does not overinterpret EBIA scores, does not imply causal relationships, and does not inadvertently discount participants' lived material experiences.

2. ***Comment:*** *EBIA scoring. Households with children receive a 0-14 scale, whereas households without children receive a 0-8 scale. The author create categories to make categories of food (in)security comparable between household with an without children. Is this new variable (categorical EBIA) treated ordinally in the analyses. Please provide additional information on this. The locally adapted instrument (EBIA) also requires more detail in the main text: adaptation process, languages used, interviewer administration, and compatibility of the EBIA recall period with the six-month network recall.*

Response: We have expanded both the Methods and Discussion sections to address each point:
First, we clarified the analytical treatment of the EBIA variable. Because households with children receive a 14-item scale and households without children receive an 8-item scale, we used the standard EBIA categorical classifications (Food Secure, Light, Moderate, Severe

Food Insecurity) to create a comparable variable across household types. We now explicitly state in the Methods that this categorical EBIA variable was treated as an ordinal outcome in all regression models (see **Lines 259-271**).

Second, we added detail on the locally adapted EBIA instrument (see **Lines 259-271**), including:

- the adaptation process based on preliminary ethnographic work and community consultation;
- the languages used (Portuguese, with clarification in Nheengatu when requested),
- interviewer administration by trained research assistants;
  and
- the specific wording adjustments made to improve comprehension while preserving item intent.

Third, we addressed the reviewer's question about recall-period compatibility. We now note that EBIA's 30-day recall period differs from the six-month recall used in the network interview, and we explain that this approach is common in mixed-methods food-security research because EBIA captures recent household experiences, whereas network data reflect longer-term patterns of exchange and support (see **Lines 282-287**).

Together, these revisions provide a clearer and more transparent account of EBIA scoring, adaptation, and analytical treatment, and they ensure that readers understand how the instrument was used and interpreted within the context of this study.

3. ***Comment:*** *Address recruitment and representativeness. The 106 households were recruited through telephone lists supplied by leaders, WhatsApp, and community meetings; 86% of respondents were women. This is common in anthropological research, but this issue should be at least briefly mentioned and discussed. No response rate, refusal rate, recruitment route breakdown, or comparison with the community census is given. The researchers should acknowledge that this strategy may preferentially enroll households already connected to leaders and community institutions, which may be the very connectivity being tested. I suggest to at least explain respondent selection within households and discuss the likely gendered knowledge and labor around food provisioning.*

   Response: We have expanded the Methods and Discussion sections to address each of these points:

   First, we now explicitly describe the recruitment routes used in the study, including leader-provided telephone lists, WhatsApp contact, and community-center meetings, and acknowledge that this strategy may preferentially enroll households already connected to community leadership or institutions. We note that this potential bias is important given that social connectivity is one of the constructs under study (see **Lines 241-246**).

Second, we added a statement clarifying that response and refusal rates were not systematically recorded, and we acknowledge this as a limitation. We also note that the sample represents 28 of the 34 ethnic groups documented in the community census, but that the recruitment approach may not fully capture households with weaker institutional ties. We also now explain respondent selection within households: interviews were conducted with the adult most knowledgeable about household food provisioning and social support, which in practice resulted in a sample that was 86% women. In the Discussion, we added a brief paragraph noting that this gender distribution reflects well-documented patterns of gendered labor and knowledge around food provisioning in Amazonian and global household-food-security research (see **Lines 241–248**).

Together, these additions provide a clearer account of recruitment procedures, potential biases, respondent selection, and the gendered nature of food-related decision-making, directly addressing the reviewer's concerns about representativeness and interpretation.

4. ***Comment:*** *Improve statistical reporting. With n=106 and many predictors, overfitting is a concern. Report coefficients and credible intervals in a main-text table rather than relying almost entirely on direction-of-effect plots. Terms such as "clear effect" should be replaced with estimates and uncertainty.*

   Response: To address this concern, we added a main-text table presenting the posterior medians and 89% credible intervals for all predictors included in the EBIA model (see **Lines 389-393**). This table allows readers to evaluate the magnitude and uncertainty of each association directly, rather than relying solely on coefficient plots or direction-of-effect summaries.

   Finally, we note in the Methods that model complexity was evaluated relative to sample size and that multicollinearity was not a concern (VIFs 1.00–1.53) (see **Lines 310-311, 387–388**). Together, these revisions improve transparency, reduce the risk of overinterpretation, and align the manuscript with best practices for statistical reporting in Bayesian regression analyses.

5. ***Comment****: Deepen the anthropological interpretation. The social-capital/economic-capital opposition is too binary. Cash income and Bolsa Família may enable sharing; however, sharing may also involve obligation, demand, hierarchy, gendered labor, conflict, or reputational pressure (as well as trust and belonging). The discussion would benefit from more ethnographic evidence and/or participant voices, distinguishing kin, neighbors, association members, and parentes, as well as the types and quantities of foods exchanged (if this information exist). I suggest avoiding broad formulations about "traditional" or "Non-western" populations and a rural-urban transition that risks essentializing 28 groups. Structural conditions (i.e. transport, land access, discrimination, employment precarity, food prices, and public services) should remain central in the analysis.*

Response: We revised the Discussion to remove binary contrasts between social and economic capital and now emphasize that these forms of capital interact in context-specific ways. We added ethnographic detail to illustrate how cash income and Bolsa Família can enable sharing, while also noting that sharing may involve obligation, gendered labor, reputational pressure, and expectations of reciprocity. This revision acknowledges that social exchange is shaped by both supportive and demanding dynamics (see **Lines 444-471**).

To incorporate participant voices and ecological specificity, we added qualitative detail describing the types of foods exchanged, including prepared meals, staple ingredients, hunted and fished items, small-scale foraged foods, and garden produce such as bananas, *macaxeira (Manihot esculenta Crantz)*, herbs used in teas, and *cheiro-verde (*a mix of parsley and scallions). We also clarified distinctions among kin, neighbors, association members, and parentes where relevant, while avoiding broad formulations about "traditional" or "non-Western" populations or rural-urban transitions that could essentialize the 28 ethnic groups represented in the sample (see **Lines 24–27, 152-163, 576-577**).

Finally, we strengthened the political-economic framing by expanding the paragraph on structural conditions. We now explicitly discuss transport limitations, land access constraints, discrimination, employment precarity, food prices, and the uneven availability of public services (see **Lines 444-460**). These additions ensure that structural conditions remain central to the interpretation of social networks and food access, as the reviewer recommended.

6. ***Comment****: Reconsider open-data claims and community governance. An unrestricted dataset containing relational structure, ethnicity or region, household characteristics, and coded network positions may be re-identifiable in a small community. The authors should state whether participants and community authorities consented specifically to open network-data release, describe de-identification, and consider controlled access. The manuscript should also report community-level authorization, how preliminary focus groups changed the research design, local assistants' intellectual contributions, and how findings and benefits will be returned to the communities.*

   Response: We have substantially expanded the Methods and Ethics sections to address each of these points.

   First, we clarified that participants and community leadership did not consent to unrestricted public release of network data, given the small size of the community and the potential for re-identification through relational structure, ethnicity, household characteristics, or network positions. We now state that the dataset will be made available only in fully de-identified, aggregate form, and that controlled access will be used for any relational data requiring additional protection. Additionally, we expanded the description of de-identification procedures, noting that all personal names, household identifiers, and relational information that could reasonably reveal identity were removed at the point of transcription, and that all nodes were represented using randomized alphanumeric codes. We also clarified that network

visualizations were generated solely from these de-identified datasets. We further added a paragraph describing community-level authorization. We now report that the study was approved by Parque das Tribos leadership, that preliminary focus groups informed the research design, and that these discussions led to adjustments in question wording, interview sequencing, and the inclusion of specific food-exchange categories requested by participants (see **Lines 225–230**, **461-471**).

Second, we acknowledge the intellectual contributions of local research assistants, who provided linguistic interpretation, cultural guidance, and assistance in adapting EBIA items for clarity. Their role is now explicitly recognized in the Methods and Acknowledgments (see **Lines 259-271, 612-629**).

Finally, we added a statement describing how findings and benefits will be returned to the community, including planned presentations to leadership, distribution of a Portuguese-language summary of results, and availability of visualizations and aggregate findings for use in local advocacy and planning (see **Lines 225-234**).

Together, these revisions strengthen the manuscript's attention to Indigenous data sovereignty, ethical data handling, community governance, and reciprocal benefit, directly addressing the reviewer's concerns.

7. ***Comment**: Presentation - Figure 1 appears inconsistent: the graphic is titled "Ego59's Network," while the caption describes the bridge between Ego36 and Ego50. Several network labels are illegible, and plotting detailed topology may increase disclosure risk. Tables and references also require careful proofreading.*

   Response: We have corrected the inconsistencies in Figure 1 by ensuring that the graphic title, caption, and described bridge-tie example now refer to the same ego and network configuration (see **Lines 132-140**). Several node labels were enlarged or removed to improve legibility while reducing disclosure risk, and the figure was simplified to avoid displaying unnecessary relational detail that could increase re-identification concerns in a small community.

   In addition, we conducted a thorough proofreading of all tables, captions, and references to ensure consistency in formatting, terminology, and citation style. These revisions improve clarity, reduce disclosure risk, and align the presentation with journal standards.

8. ***Comment**: Others:*

- *Line 124 : Sentence is the entire paragraph. This should go with following paragraph.*
- *Lines 127-129: Unnecessary wordiness make this sentence confusing. Simplify to make it more understandable.*

Response: Thank you for noting these presentation issues. We have merged the isolated sentence at Line 124 into the following paragraph to improve flow and eliminate the one-sentence paragraph (see **Lines 122–130**). We also simplified the wording in Lines 127–129 to enhance clarity and readability. These revisions resolve the structural and stylistic concerns the reviewer identified (see **Lines 126–130**).

We remain at your disposal for any additional clarifications and thank the editor and reviewers again for their thoughtful engagement with our work.

Harold Martin Wright III, PhD
Corresponding Author
