# Supplementary material for: Food sharing networks in a multi-ethnic, predominantly indigenous peri-urban community in the Brazilian Amazon
Source: PLoS One. 2026 Nov 11;21(11):e0360629. doi: 10.1371/journal.pone.0360629 (PMC13754111; doi:10.1371/journal.pone.0360629)
Supplement: Peer review history

# Peer review: Decision Letter 0

**DOI:** 10.1371/journal.pone.0360629.r001

**Authors:** Luisa Diele-Viegas

18 Mar 2026

Dear Dr. Wright III,

Thank you for submitting your manuscript to PLOS ONE. After careful consideration, we feel that it has merit but does not fully meet PLOS ONE’s publication criteria as it currently stands. Therefore, we invite you to submit a revised version of the manuscript that addresses the points raised during the review process.

Please submit your revised manuscript by May 02 2026 11:59PM. If you will need more time than this to complete your revisions, please reply to this message or contact the journal office at plosone@plos.org. When you're ready to submit your revision, log on to https://www.editorialmanager.com/pone/ and select the 'Submissions Needing Revision' folder to locate your manuscript file.

- A letter that responds to each point raised by the academic editor and reviewer(s). You should upload this letter as a separate file labeled 'Response to Reviewers'.
- A marked-up copy of your manuscript that highlights changes made to the original version. You should upload this as a separate file labeled 'Revised Manuscript with Track Changes'.
- An unmarked version of your revised paper without tracked changes. You should upload this as a separate file labeled 'Manuscript'.

If you would like to make changes to your financial disclosure, please include your updated statement in your cover letter. Guidelines for resubmitting your figure files are available below the reviewer comments at the end of this letter.

If applicable, we recommend that you deposit your laboratory protocols in protocols.io to enhance the reproducibility of your results. Protocols.io assigns your protocol its own identifier (DOI) so that it can be cited independently in the future. For instructions see: https://journals.plos.org/plosone/s/submission-guidelines#loc-laboratory-protocols. Additionally, PLOS ONE offers an option for publishing peer-reviewed Lab Protocol articles, which describe protocols hosted on protocols.io. Read more information on sharing protocols at https://plos.org/protocols?utm_medium=editorial-email&utm_source=authorletters&utm_campaign=protocols.

We look forward to receiving your revised manuscript.

Kind regards,

Luisa Maria Diele-Viegas, Ph. D.

Academic Editor

PLOS One

Journal Requirements:

When submitting your revision, we need you to address these additional requirements.

1. Please ensure that your manuscript meets PLOS ONE's style requirements, including those for file naming. The PLOS ONE style templates can be found at

https://journals.plos.org/plosone/s/file?id=wjVg/PLOSOne_formatting_sample_main_body.pdf and https://journals.plos.org/plosone/s/file?id=ba62/PLOSOne_formatting_sample_title_authors_affiliations.pdf

2. Please include a complete copy of PLOS’ questionnaire on inclusivity in global research in your revised manuscript. Our policy for research in this area aims to improve transparency in the reporting of research performed outside of researchers’ own country or community. The policy applies to researchers who have travelled to a different country to conduct research, research with Indigenous populations or their lands, and research on cultural artefacts. The questionnaire can also be requested at the journal’s discretion for any other submissions, even if these conditions are not met.  Please find more information on the policy and a link to download a blank copy of the questionnaire here: https://journals.plos.org/plosone/s/best-practices-in-research-reporting. Please upload a completed version of your questionnaire as Supporting Information when you resubmit your manuscript.

3. We note that the grant information you provided in the ‘Funding Information’ and ‘Financial Disclosure’ sections do not match.

When you resubmit, please ensure that you provide the correct grant numbers for the awards you received for your study in the ‘Funding Information’ section.

4. Please amend your authorship list in your manuscript file to include author Harold Martin Wright III, Barbara A. Piperata, Sean S. Downey, Shane A. Scaggs.

5. Please amend the manuscript submission data (via Edit Submission) to include author HM Wright III, BA Piperata, SS Downey, and SA Scaggs.

6. If the reviewer comments include a recommendation to cite specific previously published works, please review and evaluate these publications to determine whether they are relevant and should be cited. There is no requirement to cite these works unless the editor has indicated otherwise.

[Note: HTML markup is below. Please do not edit.]

Reviewers' comments:

Reviewer's Responses to Questions

**Comments to the Author**

1. Is the manuscript technically sound, and do the data support the conclusions?

Reviewer #1: Yes

Reviewer #2: Partly

**********

2. Has the statistical analysis been performed appropriately and rigorously?

Reviewer #1: Yes

Reviewer #2: N/A

**********

3. Have the authors made all data underlying the findings in their manuscript fully available?

The PLOS Data policy

Reviewer #1: Yes

Reviewer #2: Yes

**********

4. Is the manuscript presented in an intelligible fashion and written in standard English?

Reviewer #1: Yes

Reviewer #2: Yes

**********

Reviewer #1: This article is highly relevant and has strong potential to inform the development of public policies in this territory and beyond. The manuscript is clearly written, employs a robust methodology, and presents results that are consistent with real-world conditions.

Reviewer #2: Dear Authors,

During the review of the manuscript, I identified inconsistencies between the information presented in the manuscript and the data contained in the CONEP protocol (Case No. 3.687.909) submitted by you.

In particular, I observed that:

- The name of the “principal investigator” and/or the “public contact” registered with CONEP does not match any of the manuscript’s authors.

- The data collection dates described in the manuscript occur after the period authorized in the protocol registered on Plataforma Brasil.

To ensure ethical compliance and proper alignment between the study presented and the approved project, I kindly request the following clarifications:

- How the study described in the manuscript relates to the approved protocol whose number was provided.

- What role the authors played in the approved project, considering that their names do not appear as the principal investigator or public contact.

- How the discrepancy between the data collection period described in the manuscript and the period authorized by the CEP/CONEP is explained.

Additionally, I request the submission of supplementary documentation, such as:

- a clarification letter signed by the principal investigator listed in the protocol;

- proof that the authors were part of the approved research team;

- an amendment approved by the CEP/CONEP including new team members and extending the data collection period.

This information is necessary to confirm that the study presented is duly covered by the ethical approval referenced.

Thank you in advance for your clarifications.

**********

what does this mean?). If published, this will include your full peer review and any attached files.

If you choose “no”, your identity will remain anonymous but your review may still be made public.

**Do you want your identity to be public for this peer review?**  For information about this choice, including consent withdrawal, please see our Privacy Policy

Reviewer #1: No

Reviewer #2: No

**********

[NOTE: If reviewer comments were submitted as an attachment file, they will be attached to this email and accessible via the submission site. Please log into your account, locate the manuscript record, and check for the action link "View Attachments". If this link does not appear, there are no attachment files.]

To ensure your figures meet our technical requirements, please review our figure guidelines: https://journals.plos.org/plosone/s/figures

You may also use PLOS’s free figure tool, NAAS, to help you prepare publication quality figures: https://journals.plos.org/plosone/s/figures#loc-tools-for-figure-preparation.

NAAS will assess whether your figures meet our technical requirements by comparing each figure against our figure specifications.

# Author comment: Author response to Decision Letter 1

**DOI:** 10.1371/journal.pone.0360629.r002

28 May 2026

Editor comments (alterations to the manuscript are highlighted in yellow):

1. Please ensure that your manuscript meets PLOS ONE's style requirements, including those for file naming.

Response: We have ensured that the document adheres to the PLOS ONE style requirements, as indicated in the corresponding style templates.

2. Please include a complete copy of PLOS’ questionnaire on inclusivity in global research in your revised manuscript. Our policy for research in this area aims to improve transparency in the reporting of research performed outside of researchers’ own country or community. The policy applies to researchers who have travelled to a different country to conduct research, research with Indigenous populations or their lands, and research on cultural artefacts. The questionnaire can also be requested at the journal’s discretion for any other submissions, even if these conditions are not met..

Response: We have completed the required questionnaire and have included it as a supplementary file with the manuscript, a PDF copy of which is additionally included as an annex to this letter.

3. We note that the grant information you provided in the ‘Funding Information’ and ‘Financial Disclosure’ sections do not match. When you resubmit, please ensure that you provide the correct grant numbers for the awards you received for your study in the ‘Funding Information’ section..

Response: We have cross-checked the grant numbers and made the corrections, as indicated in the “Funding Information” section.

4. Please amend your authorship list in your manuscript file to include author Harold Martin Wright III, Barbara A. Piperata, Sean S. Downey, Shane A. Scaggs.

Response: We have amended the authorship list in the manuscript file as indicated, as well as including the name of Dr. Therezinha de Jesus Pinto Fraxe, as requested by a reviewer.

5. Please amend the manuscript submission data (via Edit Submission) to include author HM Wright III, BA Piperata, SS Downey, and SA Scaggs.

Response: We have amended the manuscript submission data as indicated, as well as including the name of TP Fraxe, as requested by a reviewer.

6. If the reviewer comments include a recommendation to cite specific previously published works, please review and evaluate these publications to determine whether they are relevant and should be cited. There is no requirement to cite these works unless the editor has indicated otherwise.

Response: We have amended the authorship list in the manuscript file as indicated, as well as including the name of TP Fraxe, as requested by a reviewer.

Reviewer 1 (alterations to the manuscript are in blue)

(No alterations were indicated by the reviewer).

Reviewer 2 (alterations to the manuscript are in blue):

1. The name of the “principal investigator” and/or the “public contact” registered with CONEP does not match any of the manuscript’s authors.

Response: The principal investigator and public contact, Dr. Therezinha de Jesus Pinto Fraxe of the Federal University of Amazonas (UFAM) is associated with the macro project entitled “C&T AGROFLORESTA: Uso de tecnologias para a redução do desmatamento e inclusão socioprodutiva de comunidades rurais em situação de vulnerabilidade socioeconômica,” under which this project is included, has reviewed the article in question and has accepted inclusion as co-author.

2. The data collection dates described in the manuscript occur after the period authorized in the protocol registered on Plataforma Brasil.

Response: The project was originally included in the CAAE and protocol indicated in the draft of the article with the original intent to complete fieldwork in 2021. However, due to the COVID-19 pandemic, it was transferred to the project indicated in the updated draft with a copy of the authorization and letter of clarification included as a supplementary file; however, the original CAAE and protocol were inadvertently included in the original draft due to an oversight by the lead author. It has since been updated, with the accurate chronology of interviews related to this article as substantiated by the annexed letters and highlighted in the text.

3. To ensure ethical compliance and proper alignment between the study presented and the approved project, I kindly request the following clarifications:

How the study described in the manuscript relates to the approved protocol whose number was provided.

Response: The study relates to the current macro project indicated in the current manuscript in that it investigates land resource use and social inclusion as a means of sustainable land resource use and social inclusion by Indigenous and riparian migrant groups living on the urban periphery of that Amazonian city.

What role the authors played in the approved project, considering that their names do not appear as the principal investigator or public contact.

Response: The authors fulfilled the following roles:

a. Conceptualization: Harold Wright, Sean Downey, Barbara Piperata

b. Data curation: Harold Wright

c. Formal analysis: Harold Wright , Shane Scaggs

d. Funding acquisition: Harold Wright

e. Investigation: Harold Wright, Therezinha de Jesus Pinto Fraxe

f. Methodology: Harold Wright, Barbara Piperata, Sean Downey

g. Visualization: Harold Wright, Shane Scaggs

h. Writing – original draft: Harold Wright

i. Writing – review and editing: Harold Wright, Barbara Piperata, Sean Downey, Shane Scaggs, Therezinha de Jesus Pinto Fraxe

How the discrepancy between the data collection period described in the manuscript and the period authorized by the CEP/CONEP is explained.

Response: The project was originally included in the CAAE and protocol indicated in the draft of the article with the original intent to complete fieldwork in 2021. However, due to the COVID-19 pandemic, it was transferred to the project indicated in the updated draft; however, the original CAAE and protocol were inadvertently included in the original draft due to an oversight by the lead author. The ethical approval has since been updated, as substantiated by the annexed letters.

Additionally, I request the submission of supplementary documentation, such as: a clarification letter signed by the principal investigator listed in the protocol; proof that the authors were part of the approved research team; an amendment approved by the CEP/CONEP including new team members and extending the data collection period.

Response: The requested supplementary documentation is included in the annexes attached to this letter.

Attachment Submitted filename: Responses_Plos_One_Complete.pdf

# Peer review: Decision Letter 1

**DOI:** 10.1371/journal.pone.0360629.r003

**Authors:** Luisa Diele-Viegas

2 Aug 2026

Dear Dr. Wright III,

Thank you for submitting your manuscript to PLOS ONE. After careful consideration, we feel that it has merit but does not fully meet PLOS ONE’s publication criteria as it currently stands. Therefore, we invite you to submit a revised version of the manuscript that addresses the points raised during the review process.

Dear Authors,

Thank you for submitting your revised manuscript. This second round of review shows clear and substantial improvements compared to your initial submission, and I commend you on your responsiveness to the previous feedback. While the manuscript is now much stronger and approaching publication, please address the following final points:

- Ensure all claims regarding food sharing and food insecurity are framed strictly as cross-sectional associations and clarify the analytical treatment of the scaled EBIA variable.
- Verify that recruitment biases, response/refusal rates, local instrument adaptations, and VIF values are fully reported.
- Avoid oversimplified binaries between social and economic capital, and ensure structural conditions remain central.
- Confirm that de-identification procedures, participant consent for data release, and community-level authorizations are explicitly addressed to mitigate re-identification risks in small communities.
- Fix the inconsistencies in Figure 1, merge the isolated sentence at Line 124, and simplify Lines 127–129.

==============================

Please submit your revised manuscript by Sep 16 2026 11:59PM. If you will need more time than this to complete your revisions, please reply to this message or contact the journal office at plosone@plos.org. When you're ready to submit your revision, log on to https://www.editorialmanager.com/pone/ and select the 'Submissions Needing Revision' folder to locate your manuscript file.

- A letter that responds to each point raised by the academic editor and reviewer(s). You should upload this letter as a separate file labeled 'Response to Reviewers'.
- A marked-up copy of your manuscript that highlights changes made to the original version. You should upload this as a separate file labeled 'Revised Manuscript with Track Changes'.
- An unmarked version of your revised paper without tracked changes. You should upload this as a separate file labeled 'Manuscript'.

If you would like to make changes to your financial disclosure, please include your updated statement in your cover letter. Guidelines for resubmitting your figure files are available below the reviewer comments at the end of this letter.

If applicable, we recommend that you deposit your laboratory protocols in protocols.io to enhance the reproducibility of your results. Protocols.io assigns your protocol its own identifier (DOI) so that it can be cited independently in the future. For instructions see: https://journals.plos.org/plosone/s/submission-guidelines#loc-laboratory-protocols. Additionally, PLOS ONE offers an option for publishing peer-reviewed Lab Protocol articles, which describe protocols hosted on protocols.io. Read more information on sharing protocols at https://plos.org/protocols?utm_medium=editorial-email&utm_source=authorletters&utm_campaign=protocols.

As the corresponding author, your ORCID iD is verified in the submission system and will appear in the published article. PLOS supports the use of ORCID, and we encourage all coauthors to register for an ORCID iD and use it as well. Please encourage your coauthors to verify their ORCID iD within the submission system before final acceptance, as unverified ORCID iDs will not appear in the published article. *Only*  the individual author can complete the verification step; PLOS staff *cannot*  verify ORCID iDs on behalf of authors.

We look forward to receiving your revised manuscript.

Kind regards,

Luisa Maria Diele-Viegas, Ph. D.

Academic Editor

PLOS One

Journal Requirements:

If the reviewer comments include a recommendation to cite specific previously published works, please review and evaluate these publications to determine whether they are relevant and should be cited. There is no requirement to cite these works unless the editor has indicated otherwise.

Please review your reference list to ensure that it is complete and correct. If you have cited papers that have been retracted, please include the rationale for doing so in the manuscript text, or remove these references and replace them with relevant current references. Any changes to the reference list should be mentioned in the rebuttal letter that accompanies your revised manuscript. If you need to cite a retracted article, indicate the article’s retracted status in the References list and also include a citation and full reference for the retraction notice.

[Note: HTML markup is below. Please do not edit.]

Reviewers' comments:

Reviewer's Responses to Questions

**Comments to the Author**

Reviewer #3: (No Response)

Reviewer #4: (No Response)

**********

2. Is the manuscript technically sound, and do the data support the conclusions?

Reviewer #3: Yes

Reviewer #4: Partly

**********

3. Has the statistical analysis been performed appropriately and rigorously?

Reviewer #3: Yes

Reviewer #4: Yes

**********

4. Have the authors made all data underlying the findings in their manuscript fully available?

The PLOS Data policy

Reviewer #3: Yes

Reviewer #4: Yes

**********

5. Is the manuscript presented in an intelligible fashion and written in standard English?

Reviewer #3: Yes

Reviewer #4: Yes

**********

Reviewer #3: The manuscript presents a highly relevant and robust analysis of food sharing networks and their impact on food security in a multi-ethnic, peri-urban Amazonian context. The integration of complex socioeconomic variables with hierarchical Bayesian modeling provides valuable, data-driven insights into this dynamic. I commend the authors on a methodologically sound and well-structured study. To further refine the manuscript, I have outlined a short list of minor suggestions below for your consideration:

Explicit Hypotheses: The introduction builds an excellent theoretical foundation, but the specific hypotheses being tested remain somewhat implicit. I suggest formally declaring the working hypotheses immediately following the three research questions, as this might provide a sharper and more direct analytical roadmap for the reader.

Ecological Context of Bridge Ties: The explanation that bridge ties facilitate indirect access to resources is statistically robust. However, I suggest adding one or two qualitative sentences in the discussion regarding what specific resources (e.g., hunted, fished, and foraged items versus cash-purchased goods) typically flow through these interethnic networks. Please consider this possibility, as it could deeply enrich the biological and social interpretation of the network's "quality."

VIF Clarification: In the Data Analysis section, the authors correctly mention checking for multicollinearity by calculating the Variance Inflation Factor (VIF). I suggest briefly stating the highest observed VIF value in the text, just to help explicitly confirm for the reader that multicollinearity remained strictly within acceptable limits for the modeling.

Reviewer #4: Overall, this is a promising study, but the conclusions should be narrowed and the EBIA, network, sampling, statistical, and Indigenous data-governance issues addressed.

Main revisions:

1. Distinguish food insecurity, nutrition, and “perception.” EBIA measures reported household experiences of food insecurity; it does not measure dietary quality, nutrient adequacy, anthropometric status, or access to culturally preferred foods. The manuscript repeatedly moves from EBIA scores to claims that networks “improve,” “mitigate,” or “compensate for” food insecurity and concludes that social capital is more important than economic capital. These causal and comparative claims are not justified. I suggest to reframe these throughout the manuscript as cross-sectional associations with reported household food insecurity. Also, I’d suggest a further discussion on EBIA as a tool. Is EBIA only measuring “perception” ? Otherwise, the researchers may inadvertently discount participants’ lived material experiences.

2. EBIA scoring. Households with children receive a 0–14 scale, whereas households without children receive a 0–8 scale. The author create categories to make categories of food (in)security comparable between household with an without children. Is this new variable (categorical EBIA) treated ordinally in the analyses. Please provide additional information on this. The locally adapted instrument (EBIA) also requires more detail in the main text: adaptation process, languages used, interviewer administration, and compatibility of the EBIA recall period with the six-month network recall.

3. Address recruitment and representativeness. The 106 households were recruited through telephone lists supplied by leaders, WhatsApp, and community meetings; 86% of respondents were women. This is common in anthropological research, but this issue should be at least briefly mentioned and discussed. No response rate, refusal rate, recruitment-route breakdown, or comparison with the community census is given. The researchers should acknowledge that this strategy may preferentially enroll households already connected to leaders and community institutions, which may be the very connectivity being tested. I suggest to at least explain respondent selection within households and discuss the likely gendered knowledge and labor around food provisioning.

4. Improve statistical reporting. With n=106 and many predictors, overfitting is a concern. Report coefficients and credible intervals in a main-text table rather than relying almost entirely on direction-of-effect plots. Terms such as “clear effect” should be replaced with estimates and uncertainty.

5. Deepen the anthropological interpretation. The social-capital/economic-capital opposition is too binary. Cash income and Bolsa Família may enable sharing; however, sharing may also involve obligation, demand, hierarchy, gendered labor, conflict, or reputational pressure (as well as trust and belonging). The discussion would benefit from more ethnographic evidence and/or participant voices, distinguishing kin, neighbors, association members, and parentes, as well as the types and quantities of foods exchanged (if this information exist). I suggest avoiding broad formulations about “traditional” or “Non-western” populations and a rural–urban transition that risks essentializing 28 groups. Structural conditions (i.e. transport, land access, discrimination, employment precarity, food prices, and public services) should remain central in the analysis.

6. Reconsider open-data claims and community governance. An unrestricted dataset containing relational structure, ethnicity or region, household characteristics, and coded network positions may be re-identifiable in a small community. The authors should state whether participants and community authorities consented specifically to open network-data release, describe de-identification, and consider controlled access. The manuscript should also report community-level authorization, how preliminary focus groups changed the research design, local assistants’ intellectual contributions, and how findings and benefits will be returned to the communities.

Presentation

Figure 1 appears inconsistent: the graphic is titled “Ego59’s Network,” while the caption describes the bridge between Ego36 and Ego50. Several network labels are illegible, and plotting detailed topology may increase disclosure risk. Tables and references also require careful proofreading.

Others.

Line 124 : Sentence is the entire paragraph. This should go with following paragraph.

Lines 127-129: Unnecessary wordiness make this sentence confusing. Simplify to make it more understandable.

**********

what does this mean?). If published, this will include your full peer review and any attached files.

If you choose “no”, your identity will remain anonymous but your review may still be made public.

**Do you want your identity to be public for this peer review?**  For information about this choice, including consent withdrawal, please see our Privacy Policy

Reviewer #3: **Yes:**  ANDRE LUIZ SOARES NUNES

Reviewer #4: No

**********

[NOTE: If reviewer comments were submitted as an attachment file, they will be attached to this email and accessible via the submission site. Please log into your account, locate the manuscript record, and check for the action link "View Attachments". If this link does not appear, there are no attachment files.]

To ensure your figures meet our technical requirements, please review our figure guidelines: https://journals.plos.org/plosone/s/figures

You may also use PLOS’s free figure tool, NAAS, to help you prepare publication quality figures: https://journals.plos.org/plosone/s/figures#loc-tools-for-figure-preparation.

NAAS will assess whether your figures meet our technical requirements by comparing each figure against our figure specifications.

# Author comment: Author response to Decision Letter 2

**DOI:** 10.1371/journal.pone.0360629.r004

16 Sep 2026

Editor comments:

1. Please address the following points:

� Comment: Ensure all claims regarding food sharing and food insecurity are framed strictly as cross-sectional associations and clarify the analytical treatment of the scaled EBIA variable.

� Response: All statements linking food sharing, social network characteristics, and household food insecurity have been revised to ensure they are framed strictly as cross-sectional associations. Causal language has been removed throughout. We also clarified the analytical treatment of the EBIA variable in both the Methods and Discussion sections, including a statement noting that EBIA measures reported household experiences of food insecurity rather than dietary quality or nutritional status.

� Comment: Verify that recruitment biases, response/refusal rates, local instrument adaptations, and VIF values are fully reported.

� Response: Recruitment biases and gendered sampling patterns are now explicitly described. Response/refusal rate information is reported in S2 File and referenced in the text. Local EBIA adaptations are detailed in the Methods. VIF values are reported in the Results.

� Comment: Avoid oversimplified binaries between social and economic capital, and ensure structural conditions remain central.

� Response: We revised the Discussion to remove any binary framing between social and economic capital and now emphasize that the observed patterns represent context‑specific associations rather than contrasts.

We also added a dedicated paragraph outlining the structural conditions shaping food access in Parque das Tribos, including irregular wage labor, limited formal employment, and reliance on conditional cash‑transfer programs, to ensure that political‑economic context remains central to the interpretation of results.

� Comment: Confirm that de-identification procedures, participant consent for data release, and community-level authorizations are explicitly addressed to mitigate re-identification risks in small communities.

� Response: We have fully addressed this point. The Methods section now explicitly describes our de‑identification procedures, including the removal of names, household identifiers, and any potentially identifying relational information, as well as the use of randomized alphanumeric codes for all network nodes and visualizations.

We also clarify that participants provided informed consent for data collection and use, and that approval for the study was obtained from Parque das Tribos community leadership.

Finally, we added language noting that data were stored securely, shared only in aggregate form, and handled in accordance with principles of Indigenous data sovereignty to minimize re‑identification risks in a small community.

� Comment: Fix the inconsistencies in Figure 1, merge the isolated sentence at Line 124, and simplify Lines 127-129.

� Response: All requested corrections have been completed. The inconsistencies in Figure 1 and its caption have been fixed to ensure alignment between the text, labels, and visual elements.

The isolated sentence at Line 124 has been merged into the following paragraph to resolve the structural issue noted by the reviewer.

Lines 127–129 have been simplified for clarity and improved readability.

Reviewer 3 (alterations to the manuscript are in blue and underlined, or purple and boldened/underlined in the case of alterations that address both Reviewers’ comments)

We thank the Reviewer for these suggestions. We address each in turn as follows:

1. Comment: Explicit Hypotheses: The introduction builds an excellent theoretical foundation, but the specific hypotheses being tested remain somewhat implicit. I suggest formally declaring the working hypotheses immediately following the three research questions, as this might provide a sharper and more direct analytical roadmap for the reader.

Response: In revising the Introduction, we replaced the original research questions with explicit, formally stated hypotheses, which now appear in the location the reviewer indicated (see Lines 151–185). This revision provides a clearer analytical roadmap and makes the expectations guiding the study fully explicit. The bridge paragraph preceding the hypotheses was also refined to ensure a smooth conceptual transition.

2. Comment: Ecological Context of Bridge Ties : The explanation that bridge ties facilitate indirect access to resources is statistically robust. However, I suggest adding one or two qualitative sentences in the discussion regarding what specific resources (e.g ., hunted, fished, and foraged items versus cash-purchased goods) typically flow through these interethnic networks. Please consider this possibility, as it could deeply enrich the biological and social interpretation of the network's "quality."

Response: Qualitative responses during the network interviews indicate that the resources circulating through these ties were ecologically diverse. Participants described exchanges involving prepared meals and staple ingredients but also items obtained through hunting, fishing, and small‑scale foraging, as well as garden produce such as bananas, macaxeira (Manihot esculenta Crantz), herbs used in teas, and cheiro‑verde (a mix of parsley and scallions). These mixed flows of purchased and self‑produced foods help explain why actors occupying bridge‑tie positions reported higher network quality: they connect households to resource types that differ in origin, seasonality, and reliability (see Lines 569-577).

3. Comment: In the Data Analysis section, the authors correctly mention checking for multicollinearity by calculating the Variance Inflation Factor (VIF). I suggest briefly stating the highest observed VIF value in the text, just to help explicitly confirm for the reader that multicollinearity remained strictly within acceptable limits for the modeling.

Response: We have added the highest observed VIF value directly to the Data Analysis section to make the assessment of multicollinearity fully explicit. As noted in the Results, all VIFs fell between 1.00 and 1.53, confirming that multicollinearity was well within acceptable limits for the regression models (see Lines 310-312, 387–388).

Reviewer 4 (alterations to the manuscript are in red and boldened, or purple and boldened/underlined in the case of alterations that address both Reviewers’ comments):

1. Comment: Distinguish food insecurity, nutrition, and "perception." EBIA measures reported household experiences of food insecurity; it does not measure dietary quality, nutrient adequacy, anthropometric status, or access to culturally preferred foods. The manuscript repeatedly moves from EBIA scores to claims that networks "improve," "mitigate," or "compensate for" food insecurity and concludes that social capital is more important than economic capital. These causal and comparative claims are not justified. I suggest to reframe these throughout the manuscript as cross-sectional associations with reported household food insecurity. Also, I'd suggest a further discussion on EBIA as a tool. Is EBIA only measuring "perception" ? Otherwise, the researchers may inadvertently discount participants' lived material experiences.

Response: Throughout the manuscript, we revised all statements linking social networks, food sharing, and household food insecurity to ensure they are framed strictly as cross‑sectional associations with reported household food insecurity, as measured by the EBIA. All causal or comparative language (e.g., “improve,” “mitigate,” “compensate for,” “more important than”) has been removed (see Lines 105–108, 142–150, 417-431).

To address the reviewer’s concern regarding EBIA’s conceptual domain, we added explicit clarification in both the Methods and Discussion (see Lines 259-271, 444-471). In the Discussion, we now state:

“It is important to note that the EBIA captures reported household experiences of food insecurity rather than dietary quality, nutrient adequacy, or anthropometric status, and our interpretation reflects this distinction (20, 89, 115).”

This sentence directly addresses the distinction between food insecurity, nutrition, and perception, and clarifies that EBIA measures experiential household food insecurity, not dietary intake, nutritional status, or access to culturally preferred foods.

We also expanded the Methods section to describe how EBIA was adapted for the local context, how categorical EBIA classifications were used to construct a comparable ordinal variable across household types, and how this variable was incorporated into regression models.

Together, these revisions ensure that the manuscript does not overinterpret EBIA scores, does not imply causal relationships, and does not inadvertently discount participants’ lived material experiences.

2. Comment: EBIA scoring. Households with children receive a 0-14 scale, whereas households without children receive a 0-8 scale. The author create categories to make categories of food (in)security comparable between household with an without children. Is this new variable (categorical EBIA) treated ordinally in the analyses. Please provide additional information on this. The locally adapted instrument (EBIA) also requires more detail in the main text: adaptation process, languages used, interviewer administration, and compatibility of the EBIA recall period with the six-month network recall.

Response: We have expanded both the Methods and Discussion sections to address each point:

First, we clarified the analytical treatment of the EBIA variable. Because households with children receive a 14‑item scale and households without children receive an 8‑item scale, we used the standard EBIA categorical classifications (Food Secure, Light, Moderate, Severe Food Insecurity) to create a comparable variable across household types. We now explicitly state in the Methods that this categorical EBIA variable was treated as an ordinal outcome in all regression models (see Lines 259-271).

Second, we added detail on the locally adapted EBIA instrument (see Lines 259-271), including:

� the adaptation process based on preliminary ethnographic work and community consultation;

� the languages used (Portuguese, with clarification in Nheengatu when requested),

� interviewer administration by trained research assistants;

and

� the specific wording adjustments made to improve comprehension while preserving item intent.

Third, we addressed the reviewer’s question about recall‑period compatibility. We now note that EBIA’s 30‑day recall period differs from the six‑month recall used in the network interview, and we explain that this approach is common in mixed‑methods food‑security research because EBIA captures recent household experiences, whereas network data reflect longer‑term patterns of exchange and support (see Lines 282-287).

Together, these revisions provide a clearer and more transparent account of EBIA scoring, adaptation, and analytical treatment, and they ensure that readers understand how the instrument was used and interpreted within the context of this study.

3. Comment: Address recruitment and representativeness. The 106 households were recruited through telephone lists supplied by leaders, WhatsApp, and community meetings; 86% of respondents were women. This is common in anthropological research, but this issue should be at least briefly mentioned and discussed. No response rate, refusal rate, recruitment route breakdown, or comparison with the community census is given. The researchers should acknowledge that this strategy may preferentially enroll households already connected to leaders and community institutions, which may be the very connectivity being tested. I suggest to at least explain respondent selection within households and discuss the likely gendered knowledge and labor around food provisioning.

Response: We have expanded the Methods and Discussion sections to address each of these points:

First, we now explicitly describe the recruitment routes used in the study, including leader‑provided telephone lists, WhatsApp contact, and community‑center meetings, and acknowledge that this strategy may preferentially enroll households already connected to community leadership or institutions. We note that this potential bias is important given that social connectivity is one of the constructs under study (see Lines 241-246).

Second, we added a statement clarifying that response and refusal rates were not systematically recorded, and we acknowledge this as a limitation. We also note that the sample represents 28 of the 34 ethnic groups documented in the community census, but that the recruitment approach may not fully capture households with weaker institutional ties. We also now explain respondent selection within households: interviews were conducted with the adult most knowledgeable about household food provisioning and social support, which in practice resulted in a sample that was 86% women. In the Discussion, we added a brief paragraph noting that this gender distribution reflects well‑documented patterns of gendered labor and knowledge around food provisioning in Amazonian and global household‑food‑security research (see Lines 241–248).

Together, these additions provide a clearer account of recruitment procedures, potential biases, respondent selection, and the gendered nature of food‑related decision‑making, directly addressing the reviewer’s concerns about representativeness and interpretation.

4. Comment: Improve statistical reporting. With n=106 and many predictors, overfitting is a concern. Report coefficients and credible intervals in a main-text table rather than relying almost entirely on direction-of-effect plots. Terms such as "clear effect" should be replaced with estimates and uncertainty.

Response: To address this concern, we added a main‑text table presenting the posterior medians and 89% credible intervals for all predictors included in the EBIA model (see Lines 389-393). This table allows readers to evaluate the magnitude and uncertainty of each association directly, rather than relying solely on coefficient plots or direction‑of‑effect summaries.

Finally, we note in the Methods that model complexity was evaluated relative to sample size and that multicollinearity was not a concern (VIFs 1.00–1.53) (see Lines 310-311, 387–388). Together, these revisions improve transparency, reduce the risk of overinterpretation, and align the manuscript with best practices for statistical reporting in Bayesian regression analyses.

5. Comment: Deepen the anthropological interpretation. The social-capital/economic-capital opposition is too binary. Cash income and Bolsa Famflia may enable sharing; however, sharing may also involve obligation, demand, hierarchy, gendered labor, conflict, or reputational pressure (as well as trust and belonging). The discussion would benefit from more ethnographic evidence and/or participant voices, distinguishing kin, neighbors, association members, and parentes, as well as the types and quantities of foods exchanged (if this information exist). I suggest avoiding broad formulations about "traditional" or "Non-western" populations and a rural-urban transition that risks essentializing 28 groups. Structural conditions (i.e. transport, land access, discrimination, employment precarity, food prices, and public services) should remain central in the analysis.

Response: We revised the Discussion to remove binary contrasts between social and economic capital and now emphasize that these forms of capital interact in context‑specific ways. We added ethnographic detail to illustrate how cash income and Bolsa Família can enable sharing, while also noting that sharing may involve obligation, gendered labor, reputational pressure, and expectations of reciprocity. This revision acknowledges that social exchange is shaped by both supportive and demanding dynamics (see Lines 444-471).

To incorporate participant voices and ecological specificity, we added qualitative detail describing the types of foods exchanged, including prepared meals, staple ingredients, hunted and fished items, small‑scale foraged foods, and garden produce such as bananas, macaxeira (Manihot esculenta Crantz), herbs used in teas, and cheiro‑verde (a mix of parsley and scallions). We also clarified distinctions among kin, neighbors, association members, and parentes where relevant, while avoiding broad formulations about “traditional” or “non‑Western” populations or rural‑urban transitions that could essentialize the 28 ethnic groups represented in the sample (see Lines 24–27, 152-163, 576-577).

Finally, we strengthened the political‑economic framing by expanding the paragraph on structural conditions. We now explicitly discuss transport limitations, land access constraints, discrimination, employment precarity, food prices, and the uneven availability of public services (see Lines 444-460). These additions ensure that structural conditions remain central to the interpretation of social networks and food access, as the reviewer recommended.

6. Comment: Reconsider open-data claims and community governance. An unrestricted dataset containing relational structure, ethnicity or region, household characteristics, and coded network positions may be re-identifiable in a small community. The authors should state whether participants and community authorities consented specifically to open network-data release, describe de-identification, and consider controlled access. The manuscript should also report community-level authorization, how preliminary focus groups changed the research design, local assistants' intellectual contributions, and how findings and benefits will be returned to the communities.

Response: We have substantially expanded the Methods and Ethics sections to address each of these points.

First, we clarified that participants and community leadership did not consent to unrestricted public release of network data, given the small size of the community and the potential for re‑identification through relational structure, ethnicity, household characteristics, or network positions. We now state that the dataset will be made available only in fully de‑identified, aggregate form, and that controlled access will be used for any relational data requiring additional protection. Additionally, we expanded the description of de‑identification procedures, noting that all personal names, household identifiers, and relational information that could reasonably reveal identity were removed at the point of transcription, and that all nodes were represented using randomized alphanumeric codes. We also clarified that network visualizations were generated solely from these de‑identified datasets. We further added a paragraph describing community‑level authorization. We now report that the study was approved by Parque das Tribos leadership, that preliminary focus groups informed the research design, and that these discussions led to adjustments in question wording, interview sequencing, and the inclusion of specific food‑exchange categories requested by participants (see Lines 225–230, 461-471).

Second, we acknowledge the intellectual contributions of local research assistants, who provided linguistic interpretation, cultural guidance, and assistance in adapting EBIA items for clarity. Their role is now explicitly recognized in the Methods and Acknowledgments (see Lines 259-271, 612-629).

Finally, we added a statement describing how findings and benefits will be returned to the community, including planned presentations to leadership, distribution of a Portuguese‑language summary of results, and availability of visualizations and aggregate findings for use in local advocacy and planning (see Lines 225-234).

Together, these revisions strengthen the manuscript’s attention to Indigenous data sovereignty, ethical data handling, community governance, and reciprocal benefit, directly addressing the reviewer’s concerns.

7. Comment: Presentation - Figure 1 appears inconsistent: the graphic is titled "Ego59's Network," while the caption describes the bridge between Ego36 and Ego50. Several network labels are illegible, and plotting detailed topology may increase disclosure risk. Tables and references also require careful proofreading.

Response: We have corrected the inconsistencies in Figure 1 by ensuring that the graphic title, caption, and described bridge‑tie example now refer to the same ego and network configuration (see Lines 132-140). Several node labels were enlarged or removed to improve legibility while reducing disclosure risk, and the figure was simplified to avoid displaying unnecessary relational detail that could increase re‑identification concerns in a small community.

In addition, we conducted a thorough proofreading of all tables, captions, and references to ensure consistency in formatting, terminology, and citation style. These revisions improve clarity, reduce disclosure risk, and align the presentation with journal standards.

8. Comment: Others:

� Line 124 : Sentence is the entire paragraph. This should go with following paragraph.

� Lines 127-129: Unnecessary wordiness make this sentence confusing. Simplify to make it more understandable.

Response: Thank you for noting these presentation issues. We have merged the isolated sentence at Line 124 into the following paragraph to improve flow and eliminate the one‑sentence paragraph (see Lines 122–130). We also simplified the wording in Lines 127–129 to enhance clarity and readability. These revisions resolve the structural and stylistic concerns the reviewer identified (see Lines 126–130).

We remain at your disposal for any additional clarifications and thank the editor and reviewers again for their thoughtful engagement with our work.

Attachment Submitted filename: Responses_Plos_One_Pt2_v3.pdf

# Peer review: Decision Letter 2

**DOI:** 10.1371/journal.pone.0360629.r005

**Authors:** Luisa Diele-Viegas

30 Sep 2026

Food sharing networks in a multi-ethnic, predominantly indigenous peri-urban community in the Brazilian Amazon

PONE-D-25-54642R2

Dear Dr. Wright III,

We’re pleased to inform you that your manuscript has been judged scientifically suitable for publication and will be formally accepted for publication once it meets all outstanding technical requirements.

Within one week, you’ll receive an e-mail detailing the required amendments. When these have been addressed, you’ll receive a formal acceptance letter and your manuscript will be scheduled for publication.

An invoice will be generated when your article is formally accepted. Please note, if your institution has a publishing partnership with PLOS and your article meets the relevant criteria, all or part of your publication costs will be covered. Please make sure your user information is up-to-date by logging into Editorial Manager at Editorial Manager® and clicking the ‘Update My Information' link at the top of the page. For questions related to billing, please contact billing support.

If your institution or institutions have a press office, please notify them about your upcoming paper to help maximize its impact. If they’ll be preparing press materials, please inform our press team as soon as possible -- no later than 48 hours after receiving the formal acceptance. Your manuscript will remain under strict press embargo until 2 pm Eastern Time on the date of publication. For more information, please contact onepress@plos.org.

Kind regards,

Luisa Maria Diele-Viegas, Ph. D.

Academic Editor

PLOS One

Additional Editor Comments (optional):

Reviewers' comments:

Reviewer's Responses to Questions

**Comments to the Author**

Reviewer #3: All comments have been addressed

Reviewer #4: All comments have been addressed

**********

2. Is the manuscript technically sound, and do the data support the conclusions?

Reviewer #3: Yes

Reviewer #4: Yes

**********

3. Has the statistical analysis been performed appropriately and rigorously?

Reviewer #3: Yes

Reviewer #4: Yes

**********

4. Have the authors made all data underlying the findings in their manuscript fully available?

The PLOS Data policy

Reviewer #3: Yes

Reviewer #4: Yes

**********

5. Is the manuscript presented in an intelligible fashion and written in standard English?

Reviewer #3: Yes

Reviewer #4: (No Response)

**********

Reviewer #3: The authors have done an excellent job in this second round of review. The manuscript has been significantly improved, and all previous concerns regarding causal language, the interpretation of the EBIA metric, potential recruitment biases, and statistical transparency have been rigorously and satisfactorily resolved. The explicit inclusion of hypotheses in the introduction and the qualitative contextualization of local exchange dynamics have greatly enriched the discussion. The study is robust and ready for publication.

For the final polishing of the manuscript, I suggest only two very minor corrections aimed at facilitating methodological replication and readability for a broader audience:

Adapted Instrument Appendix: Since the Methods section has been expanded to detail the local adaptation process of the EBIA scale, including wording adjustments and the use of Portuguese and Nheengatu, it would be an excellent addition to include the exact adapted questionnaire as a Supplementary File. This will ensure that the instrument can be easily replicated by other researchers investigating food security in Indigenous and peri-urban communities in the Amazon.

Credible Interval (CI) Citation: In Table 3, the authors report posterior median effects using 89% credible intervals. As readers from the social sciences and public health may not be familiar with this approach (and might question the absence of the traditional 95% threshold), I recommend adding a brief methodological citation in the Data Analysis section to justify the 89% choice. I suggest providing both the theoretical justification for avoiding arbitrary thresholds and the computational stability rationale for MCMC simulations by citing:

McElreath, R. (2020). Statistical rethinking: A Bayesian course with examples in R and Stan (2nd ed.). CRC press.

Makowski, D., Ben-Shachar, M. S., & Lüdecke, D. (2019). bayestestR: Describing Effects and their Uncertainty, Existence and Significance within the Bayesian Framework. Journal of Open Source Software, 4(40), 1541. DOI: 10.21105/joss.01541

This is a valid and growing convention in contemporary Bayesian modeling, and a simple explanatory sentence will prevent any reader confusion.

Congratulations on the excellent work and the important contribution to understanding food security dynamics in the Amazon. I highly recommend the manuscript for acceptance after these very minor adjustments.

Reviewer #4: The authors have addressed the concerns and comments raised in my previous review. I recommend this article for publication.

**********

what does this mean?). If published, this will include your full peer review and any attached files.

If you choose “no”, your identity will remain anonymous but your review may still be made public.

**Do you want your identity to be public for this peer review?**  For information about this choice, including consent withdrawal, please see our Privacy Policy

Reviewer #3: **Yes:**  ANDRE LUIZ SOARES NUNES

Reviewer #4: **Yes:**  Catalina I. Fernández, PhD

**********

# Editor report: Acceptance letter

**DOI:** 10.1371/journal.pone.0360629.r006

**Authors:** Luisa Diele-Viegas

PONE-D-25-54642R2

PLOS One

Dear Dr. Piperata,

I'm pleased to inform you that your manuscript has been deemed suitable for publication in PLOS One. Congratulations! Your manuscript is now being handed over to our production team.

At this stage, our production department will prepare your paper for publication. This includes ensuring the following:

* All references, tables, and figures are properly cited

* All relevant supporting information is included in the manuscript submission,

* There are no issues that prevent the paper from being properly typeset

You will receive further instructions from the production team, including instructions on how to review your proof when it is ready. Please keep in mind that we are working through a large volume of accepted articles, so please give us a few days to review your paper and let you know the next and final steps.

Lastly, if your institution or institutions have a press office, please let them know about your upcoming paper now to help maximize its impact. If they'll be preparing press materials, please inform our press team within the next 48 hours. Your manuscript will remain under strict press embargo until 2 pm Eastern Time on the date of publication. For more information, please contact onepress@plos.org.

You will receive an invoice from PLOS for your publication fee after your manuscript has reached the completed accept phase. If you receive an email requesting payment before acceptance or for any other service, this may be a phishing scheme. Learn how to identify phishing emails and protect your accounts at https://explore.plos.org/phishing.

If we can help with anything else, please email us at customercare@plos.org.

Thank you for submitting your work to PLOS One and supporting open access.

Kind regards,

PLOS One Editorial Office Staff

on behalf of

Dr. Luisa Maria Diele-Viegas

Academic Editor

PLOS One
